# Cardiovascular Disease Risk Among Older Asian, Native Hawaiian, Pacific Islanders Lung Cancer Survivors

**DOI:** 10.1002/cam4.70702

**Published:** 2025-02-20

**Authors:** Yancen Pan, Chun‐Pin Esther Chang, Randa Tao, Anees Daud, Jincheng Shen, Nathan D. Wong, Roch A. Nianogo, Jianyu Rao, Thomas Varghese, Zuo‐Feng Zhang, Mia Hashibe

**Affiliations:** ^1^ Department of Epidemiology UCLA Fielding School of Public Health Los Angeles California USA; ^2^ Division of Public Health, Department of Family and Preventive Medicine University of Utah School of Medicine Salt Lake City Utah USA; ^3^ Huntsman Cancer Institute University of Utah Salt Lake City Utah USA; ^4^ Department of Radiation Oncology University of Utah School of Medicine Salt Lake City Utah USA; ^5^ Division of Cardiovascular Medicine University of Utah School of Medicine Salt Lake City Utah USA; ^6^ Division of Biostatistics, Department of Population Health Sciences University of Utah School of Medicine Salt Lake City Utah USA; ^7^ Division of Cardiology UCI Heart Disease Prevention Program Irvine California USA; ^8^ Thoracic Surgery, Department of Surgery University of Utah School of Medicine Salt Lake City Utah USA

**Keywords:** Asian, cardiovascular disease, lung cancer survivor, native Hawaiian, Pacific islander, racial disparity

## Abstract

**Background:**

There may be heterogeneity in lung cancer‐related outcomes for individuals who are Asian, Native Hawaiian, and Pacific Islanders (ANHPI).

**Objectives:**

The aims of this study were to investigate possible disparities in cardiovascular disease (CVD) risk between ANHPI and Non‐Hispanic White (NHW) lung cancer survivors and evaluate potential CVD risk factors.

**Methods:**

A total of 3920 ANHPI and 11,760 NHW lung cancer patients aged 66 years and older were identified from the Surveillance, Epidemiology, and End Results (SEER)‐Medicare registry from 1999 to 2017. Cox proportional hazards models were used to calculate adjusted hazard ratios (HRs) and 95% confidence intervals (95% CIs) for incident CVD, comparing ANHPI lung cancer patients and their race/ethnicity subgroups to NHW lung cancer patients.

**Results:**

Compared to NHW lung cancer patients, ANHPI lung cancer patients had a lower risk of developing heart failure (HR, 0.64, 95% CI, 0.53–0.76) and ischemic heart disease (HR, 0.76, 95% CI, 0.60–0.95). Additionally, compared to Chinese lung cancer patients, Pacific Islander, South Asian, and Southeast Asian lung cancer patients had a higher risk of heart failure.

**Conclusion:**

While ANHPI lung cancer patients had lower risks of heart failure and ischemic heart disease than NHW lung cancer patients, heterogeneity in risk was observed among ANHPI subgroups. Further research is needed to investigate the reasons for the higher risk of several CVDs among Pacific Islander, South Asian, and Southeast Asian lung cancer patients.

## 1 Introduction

In 2024, there were 234,580 estimated new diagnoses of lung cancer [1]. The 5‐year survival rate of lung cancer is approximately 26.6% in the United States, which is relatively low compared to most other cancers [2]. However, because of earlier detection and improved treatment, survival has been improving [1], resulting in an increasing number of lung cancer survivors in the United States [2, 3]. In 2022, there were approximately 654,620 men and women with a history of lung cancer in the United States, and this number is expected to rise due to increased lung cancer screening and advancements in treatment [4, 5].

For the Asian, Native Hawaiian, and Pacific Islander (ANHPI) population, the age‐adjusted incidence rate and mortality rate of lung cancer were 34.2 (per 100,000 population) and 19.8 (per 100,000 population), respectively. These rates are approximately half of those in the Non‐Hispanic White (NHW) population [1, 3]. However, there is large variation among ANHPI subgroups based on geographical origin, acculturation, and socioeconomic status [1, 6]. Compared with NHW lung cancer patients, ANHPI lung cancer patients had a similar 5‐year survival rate (26% in ANHPIs vs. 23% in NHWs) but were slightly more likely to be diagnosed at an advanced stage (58% in ANHPIs vs. 52% in NHWs) [1, 2]. Disparities in targeted treatment for ANHPI lung cancer patients as well as lung cancer screening in the ANHPI population have been identified [1].

As the number of lung cancer survivors increases in the UNITES STATES, the burden of other chronic medical conditions will also become more prevalent in this population. Cardiovascular disease (CVD) was the leading cause of death in the United States, responsible for 874,613 deaths in the United States in 2019 [7, 8]. Patients with lung cancer have similar risk factors such as tobacco smoking that predispose them to CVD. In addition, lung cancer treatment, including certain types of chemotherapy and radiation that might increase the risk of CVD [8]. The prevalence of heart disease was lower in Asians in the United States. compared with NHW individuals (7.7% vs. 11.5%) [9]. However, the ANHPI population is a heterogeneous group including individuals with high CVD risk, such as the South Asian population, as well as individuals at lower CVD risk, such as the East Asian population [9, 10].

Although there are several studies investigating CVD risks among lung cancer survivors [11, 12, 13, 14, 15, 16], few have focused on possible disparities in CVD among ANHPI lung cancer survivors. We hypothesize that the overall ANHPI lung cancer patients may have a lower risk of CVD compared to NHW lung cancer patients. We will use the NHW lung cancer patients as the reference group to investigate differences with a broader but defined US population. We also hypothesize that the CVD risk among ANHPI subgroups will be heterogeneous. We will use Chinese lung cancer patients as the reference group since they were the largest ANHPI sub‐group to investigate differences among the ANHPI subgroups. The aims of our study were to investigate the potential disparity of CVD incidence among ANHPI and NHW lung cancer survivors and evaluate potential risk factors for CVD among ANHPI lung cancer survivors.

## 2 Methods

The study population was from the SEER‐Medicare database. SEER‐Medicare is a linked dataset that provides information about Medicare beneficiaries with cancer. SEER provides details of clinical and demographic information for cancer patients. Medicare provides enrollment information and claims for health care services for these cancer patients [17].

**FIGURE 1:**
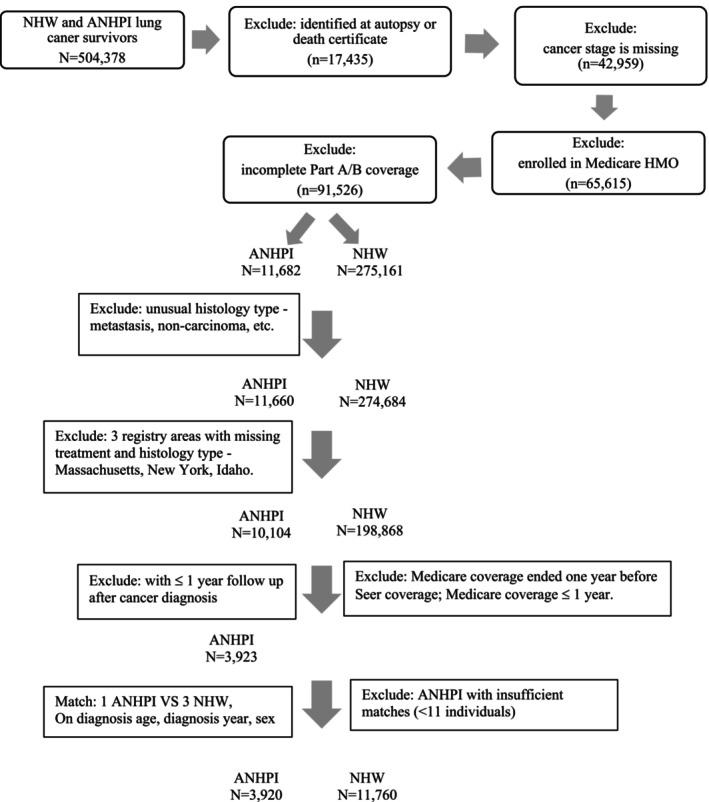
Inclusion and exclusion criteria.

We included ANHPI or NHW lung cancer patients who were 66 years and older, diagnosed with first primary invasive lung cancer (SEER code 22030, with ICD‐O‐3 behavior code = 3) between 2000 and 2017 (Figure 1). We excluded individuals who were diagnosed with lung cancer from autopsy or death certificates, as well as individuals who had cancer stage missing. Medicare claims were available since 1999. We also excluded individuals with < 1 year of claims to ensure we had sufficient data to assess baseline comorbidities. We excluded individuals who were enrolled in an HMO or who were without full coverage with Medicare Part A and Part B during the study follow‐up period. Considering the long latency period of CVD, we further excluded lung cancer patients with 1 year or less of follow‐up. Histological types that were non‐carcinoma were also excluded [18, 19]. We also excluded participants who were registered in Idaho, Massachusetts, and New York because cancer histology and treatments were not available in these three registries. An ANHPI lung cancer patient was matched to 3 NHW lung cancer patients on diagnosis age (±1 year), diagnosis year (±1 year) and sex. We identified 3920 ANHPI lung cancer patients and matched 11,760 NHW lung cancer patients to them. We obtained approval for this study from the Institutional Review Board at the University of Utah. A waiver of Informed Consent was approved.

Demographic data from SEER included diagnosis year, diagnosis age, sex, cancer registry, and rural/urban residence at cancer diagnosis. Clinical characteristics included stage, histology, grade, laterality, baseline Charlson comorbidity index (CCI), first course cancer treatment, and Medicare claims for chemotherapy and immunotherapy (by Healthcare Common Procedure Coding System (HCPCS)). Urban was defined as counties of metropolitan areas (code 0–3 of Rural–Urban Continuum) and rural was defined as cities of non‐metropolitan areas (code 4–9 of Rural–Urban Continuum). Education and income were available at the census tract level, extracted from the recent census records (Census 2000 or 2010). A modified CCI score was calculated by Physician/carrier (NCH), outpatient, and hospital (MedPAR) claims from Medicare records within 1 year before lung cancer diagnosis, excluding cancers and CVDs [20].

CVD diagnosed within 1 year after lung cancer diagnosis were excluded as incident events. CVD outcomes were also identified from NCH, outpatient and MedPAR claims. ICD‐9/10 codes for each CVD condition were identified by Chronic Conditions Warehouse (CCW) from 1999 to 2020. Three CVD conditions were identified as the outcomes of interest: heart failure, ischemic heart disease and stroke or transient ischemic attack. Patients were considered to have CVD events if the corresponding ICD code appeared in 1 claim of MedPAR, or at least 2 claims of outpatient or NCH within 30–60 days. Follow‐up time started at lung cancer diagnosis date to the first event of CVD, death, or the end of Medicare follow‐up. Death was identified in both the SEER and Medicare data.

Demographic and clinical characteristics were coded as categorical variables and compared between ANHPI and NHW groups using chi‐squared (*χ* ^2^) tests. The incidence rates of each type of CVD were calculated by different race/ethnicity groups and subgroups. We then employed Kaplan–Meier curves to visually compare CVD outcomes between ANHPI and NHW groups and to identify any serious violations of the proportional hazards assumption (Figures S1–S3). Cox proportional hazards models were then used to calculate hazard ratios (HRs) and 95% confidence intervals (95% CIs) for the difference in CVD risks among different race/ethnicity groups, using NHW lung cancer patients as the reference group. The Cox proportional models were clustered on matched pairs based on matching factors (diagnosis age, diagnosis year, sex) and adjusted for cancer registry. We also considered additional models with more extensive covariate adjustments, including socioeconomic status and lifestyle factors (see Table S4). A directed acyclic graph (DAG) is shown in Figure S4 to illustrate the relationship of various factors with our exposure (race and ethnicity) and CVD outcomes in these models. We additionally stratified by sex and restricted the analysis to patients with one primary cancer as a sensitivity analysis. Cox proportional hazard models were also used to compare the CVD risks among ANHPI subgroups, using the Chinese lung cancer patients—the largest subgroup—as the reference group, adjusting for age, year of diagnosis, sex, and cancer registry. Similarly, we also fitted models further adjusting for socioeconomic status and lifestyle factors in Table S5. To assess the potential change of the HRs over time, we also considered piecewise HRs in the Cox models, allowing the effect of race/ethnicity during 1–5 years to be different from > 5 years after lung cancer diagnosis. 1–5 years and > 5 years after lung cancer diagnosis. Furthermore, the proportional hazard assumption was formally tested by including the time‐dependent covariate in the model (race/ethnicity multiplied by follow up time, treating follow‐up time as continuous). If the assumption was not met, the flexible parametric survival model was used for outcome regression [21]. Additional Cox proportional hazards models were fitted separately for ANHPI and NHW lung cancer patients to investigate potential demographic or clinical risk factors for CVDs, with each model adjusting for potential confounders specific to each risk factor. The results were then compared between the two race/ethnicity groups.

We also performed additional sensitivity analyses to assess the impact of competing events. First, we plotted the cumulative incidence function (CIF) curves following a competing risk framework to compare the different CVD events before death of the three types of CVD events (Figures S5–S7). We then used the fine‐Gray method to estimate HRs in the primary and subgroup analysis on race/ethnicity group comparisons, accounting for death as competing on CVD risks (see Tables S8 and S9).

We used SAS 9.4 (SAS Institute Inc., Cary, NC) and also STATA 17 for the flexible parametric survival model.

## 3 Results

Among the 3920 ANHPI and 11,760 NHW lung cancer survivors, ANHPI lung cancer patients had higher education levels, higher income levels, lower baseline CCI, and lived at a higher proportion in the west and urban areas (Table 1). For clinical characteristics, ANHPI lung cancer patients had a higher proportion diagnosed with only one primary cancer and adenocarcinoma. A smaller proportion of ANHPI lung cancer patients received radiation therapy and surgery, but more of them received chemotherapy. ANHPI lung cancer patients also had slightly more immunotherapy claims. Follow‐up years and laterality were similar between ANHPI and NHW lung cancer patients. For eligible patients who survived for a year or more, there was a higher proportion diagnosed at distant stage among ANHPI lung cancer patients. When we included patients who survived for < 1 year, the stage distribution for overall lung cancer survivors was similar to the distribution in general lung cancer survivors (data not shown) [3].

**TABLE 1:** Demographic and clinical characteristics of ANHPI and NHW lung cancer survivors.

	ANHPI lung cancer survivors (*n* = 3920)	NHW lung cancer survivors (*n* = 11,760)	*p* (chi‐square)
	*n* (%)	*n* (%)	
Sex			
Male	1973 (50.33)	5919 (50.33)	1.00
Female	1947 (49.67)	5841 (49.67)	
Age at diagnosis, year			
66–70	879 (22.42)	2637 (22.42)	1.00
71–75	1103 (28.14)	3309 (28.14)	
76–80	1009 (25.74)	3027 (25.74)	
81–85	618 (15.77)	1854 (15.77)	
86+	311 (7.93)	933 (7.93)	
Diagnosis year			
2000–2005	1046 (26.68)	3138 (26.68)	1.00
2006–2010	1061 (27.07)	3183 (27.07)	
2011–2014	980 (25.00)	2939 (24.99)	
2015–2017	833 (21.25)	2500 (21.26)	
Charlson Comorbidity Index (CCI) at baseline			
0	1539 (39.26)	4108 (34.93)	< 0.001
1	1311 (33.44)	4124 (35.07)	
2+	1070 (27.30)	3528 (30.00)	
Registry area			
West	3498 (89.23)	3991 (33.94)	< 0.001
Northeast	252 (6.43)	2679 (22.78)	
Midwest	55 (1.40)	1619 (13.77)	
South	115 (2.93)	3471 (29.52)	
Follow up, year			
> 1–5	3009 (76.76)	8920 (75.85)	0.40
≥ 5–10	684 (17.45)	2165 (18.41)	
≥ 10	227 (5.79)	675 (5.74)	
Urbanization			
Urban^a^	> 3731 (> 95.18)	> 9442 (> 80.29)	< 0.001
Rural	178 (4.54)	2307 (19.62)	
Unknown	< 11 (< 0.28)^c^	< 11 (< 0.09)^c^	
Education: proportion above college^b^ (census tract)			
0%–40%	588 (15.00)	2388 (20.31)	< 0.001
> 40%–60%	1348 (34.39)	4208 (35.78)	
> 60%–80%	> 1417 (> 36.15)	3740 (31.80)	
> 80%–100%	556 (14.18)	1413 (12.02)	
Missing	< 11 (< 0.28)^c^	11 (0.09)	
Income (median income in census tract)			
≤ 40,000	830 (21.17)	3273 (27.83)	< 0.001
40,000–60,000	> 1168 (> 29.80)	3996 (33.98)	
60,000–80,000	987 (25.18)	2344 (19.93)	
> 80,000	924 (23.57)	2135 (18.15)	
Missing	< 11 (< 0.28)^c^	12 (0.10)	
Tobacco use			
Yes	617 (15.74)	3963 (33.70)	< 0.001
No	3303 (84.26)	7797 (66.30)	
Sequence number			
One primary only	3517 (89.72)	10,121 (86.06)	< 0.001
First of many primaries	403 (10.28)	1639 (13.94)	
Histology			
SCLC‐Small cell	201 (5.13)	964 (8.20)	< 0.001
NSCLC‐Adenocarcinoma	2389 (60.94)	5111 (43.46)	
NSCLC‐Squamous cell	640 (16.33)	3166 (26.92)	
NSCLC‐Large cell carcinoma	77 (1.96)	251 (2.13)	
Other NSCLC	413 (10.54)	1506 (12.81)	
Unspecific lung cancer	200 (5.10)	762 (6.48)	
Grade			
Grade I	351 (8.95)	969 (8.24)	0.001
Grade II	920 (23.47)	2637 (22.42)	
Grade III	872 (22.24)	2954 (25.12)	
Grade IV	107 (2.73)	397 (3.38)	
Not determined/stated/applicable	1670 (42.60)	4803 (40.84)	
Laterality			
Right: origin of primary	2224 (56.73)	6744 (57.35)	0.42
Left: origin of primary	1596 (40.71)	4756 (40.44)	
Others^d^	100 (2.55)	260 (2.21)	
Stage			
Localized	1179 (30.08)	4432 (37.69)	< 0.001
Regional	1128 (28.78)	3866 (32.87)	
Distant	1613 (41.15)	3462 (29.44)	
Radiation therapy			
Yes	1332 (33.98)	4650 (39.54)	< 0.001
No	2552 (65.10)	6918 (58.83)	
Unknown	36 (0.92)	192 (1.63)	
Chemotherapy			
Yes	1757 (44.82)	4599 (39.11)	< 0.001
No/unknown^e^	2163 (55.18)	7161 (60.89)	
Surgery			
Yes	> 1384 (> 35.31)	4654 (39.57)	< 0.001
No	2525 (64.41)	7045 (59.91)	
Unknown	< 11 (< 0.28)^c^	61 (0.52)	
Number of chemotherapy claims (from Medicare)			
*N* = 0	2028 (51.73)	6110 (51.96)	0.03
1 ≤ *N* ≤ 8	589 (15.03)	1538 (13.08)	
8 < *N* ≤ 14	394 (10.05)	1255 (10.67)	
14 < *N* ≤ 24	465 (11.86)	1478 (12.57)	
*N* > 24	444 (11.33)	1379 (11.73)	
Number of immunotherapy claims (from Medicare)			
*N* = 0	3624 (92.45)	11,046 (93.93)	0.005
1 ≤ *N* ≤ 3	89 (2.27)	182 (1.55)	
3 < *N* ≤ 7	79 (2.02)	181 (1.54)	
7 < *N* ≤ 15	58 (1.48)	172 (1.46)	
*N* > 15	70 (1.79)	179 (1.52)	
ANHPI subgroups			
Chinese	1042 (26.58)		
Japanese	670 (17.09)		
Filipino	652 (16.63)		
Hawaiian	188 (4.80)		
Korean	356 (9.08)		
Vietnamese	433 (11.05)		
Indian or Pakistani	143 (3.65)		
Other Southeast Asian^f^	54 (1.38)		
Pacific Islander	67 (1.71)		
Other Asian^g^	315 (8.04)		

Abbreviations: ANHPI, Asian, Native Hawaiian, and Pacific Islander; NHW, Non‐Hispanic White.

^a^ Urban is defined as counties of metropolitan areas (code 0–3 of Rural–Urban Continuum/Beale code); Rural is defined as cities of non‐metropolitan areas (code 4–9 of Rural–Urban Continuum/Beale code).

^b^ Including some college and at least 4 years of college.

^c^ Counts < 11 are not shown; Centers for Medicare & Medicaid Services (CMS) Cell Suppression Policy.

^d^ Others include: Not a paired site; Only one side involved, right or left origin unspecified; Bilateral involvement, lateral origin unknown; Stated to be single primary; Paired site.

^e^ The original dataset combined No and Unknown together for Chemotherapy.

^f^ Other Southeast Asian: including Laotian, Hmong, Kampuchean, and Thai.

^g^ Other Asian: including Asian, NOS and Oriental, NOS.

**FIGURE 2:**
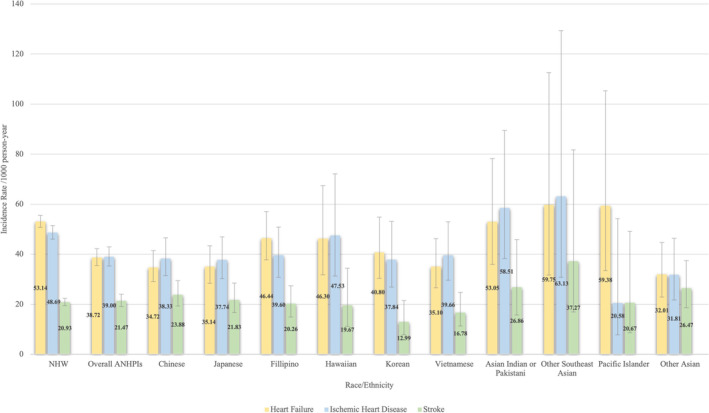
Incidence Rate and 95% confidence interval of CVD, by race/ethnicity.

The incidence rate for heart failure, ischemic heart disease, and stroke among NHW, ANHPI overall, and ANHPI subgroups is shown in Figure 2. Compared to NHW lung cancer patients, ANHPI lung cancer patients had a lower incidence of heart failure and ischemic heart disease (Table 2). Chinese, Japanese, Vietnamese, and other Asian lung cancer patients had a lower incidence of heart failure than NHW lung cancer patients. A lower incidence of ischemic heart disease among Chinese, Japanese, and other Asian lung cancer patients was also observed compared to NHW lung cancer patients. The results of CVD risks comparing ANHPI lung cancer patients with NHW lung cancer patients, stratified by sex, were shown in the Table S1.

**TABLE 2:** Risk of CVD among lung cancer patients, ANHPI and ANHPI Subgroup versus NHW.

	Heart failure		Ischemic heart disease		Stroke/transient ischemic attack	
	Cases (*N*)/total (*N*)	HR (95% CIs)^c^	Cases (*N*)/total (*N*)	HR (95% CIs)^c^	Cases (*N*)/total (*N*)	HR (95% CIs)^c^
NHW	1807/9122	1 (ref)	1208/6766	1 (ref)	820/10498	1 (ref)
Overall ANHPI	482/3348	0.64 (0.53, 0.76)	387/2748	0.76 (0.60, 0.95)	286/3573	0.97 (0.77, 1.21)
Chinese	> 117/912	0.53 (0.39, 0.72)	> 86/719	0.67 (0.45, 1.00)	> 74/965	1.04 (0.72, 1.51)
Japanese	83/587	0.48 (0.32, 0.71)	77/521	0.64 (0.41, 1.00)	53/613	0.96 (0.58, 1.59)
Filipino	86/534	0.83 (0.59, 1.17)	59/437	0.88 (0.57, 1.36)	41/578	1.02 (0.64, 1.62)
Hawaiian	26/154	0.76 (0.38, 1.51)	21/131	1.25 (0.55, 2.84)	12/175	1.14 (0.44, 2.98)
Korean	42/307	0.74 (0.46, 1.19)	32/248	0.85 (0.47, 1.54)	15/324	0.64 (0.33, 1.24)
Vietnamese	49/378	0.60 (0.40, 0.90)	44/305	0.71 (0.42, 1.23)	25/389	0.77 (0.45, 1.33)
Indian or Pakistani	24/115	0.75 (0.41, 1.36)	20/90	1.40 (0.61, 3.19)	13/126	0.89 (0.38, 2.11)
Other Southeast Asian^a^	< 11/49^d^	1.26 (0.47, 3.37)	< 11/38^d^	1.82 (0.56, 5.93)	< 11/48^d^	1.03 (0.34, 3.16)
Pacific Islander	11/48	0.93 (0.33, 2.63)	< 11/48^d^	0.39 (0.11, 1.47)	< 11/61^d^	1.02 (0.32, 3.24)
Other Asian^b^	33/264	0.54 (0.31, 0.95)	26/211	0.45 (0.21, 0.93)	31/294	1.39 (0.80, 2.43)

*Note:* Please note that cancer patient numbers differ from Table 1 because prevalent cases of CVD were excluded for each outcome of interest. For example, lung cancer patients with prevalent heart failure were excluded for the analysis of incident heart failure.

Abbreviations: ANHPI, Asian, Native Hawaiian, and Pacific Islander; CIs, Confidence Intervals; HR, Hazard Ratio; NHW, Non‐Hispanic White.

^a^ Other Southeast Asian: including Laotian, Hmong, Kampuchean, and Thai.

^b^ Other Asian: including Asian, NOS and Oriental, NOS.

^c^ COX proportional hazard model, adjusting for matched pairs and registry.

^d^ Counts < 11 are not shown, Centers for Medicare and Medicaid Services (CMS) Cell Suppression Policy.

We also compared the CVD risks among ANHPI subgroups with Chinese lung cancer patients as the reference group (Table 3). Most ANHPI subgroups had a higher risk of heart failure than Chinese lung cancer patients, including Filipino, Indian/Pakistani, other Southeast Asian, and Pacific Islander lung cancer patients. Except for a higher risk of ischemic heart disease among Indian or Pakistani lung cancer patients, we did not observe any statistically significant differences in ischemic heart disease and stroke between Chinese lung cancer patients and other ANHPI subgroups. The results of piecewise HRs of CVD stratified on follow‐up years for 1–5 years and more than 5 years of follow‐up time are shown in Table S2 for NHW patients as reference and in Table S3 for Chinese lung cancer patients as reference. The piecewise HRs for 1–5 years after lung cancer diagnosis were similar to the HRs for Cox regression in Tables 2 and 3. In > 5 years after lung cancer diagnosis, the risk of heart failure was lower in Chinese lung cancer patients, and the risk of ischemic heart disease was lower in Japanese lung cancer patients compared to NHW lung cancer patients. When compared to Chinese lung cancer patients, the risk of heart failure in Indian and Pakistani lung cancer patients was higher, and the risk of ischemic heart disease in other Asian lung cancer patients was higher in > 5 years after lung cancer diagnosis.

**TABLE 3:** The risk of CVD among lung cancer patients, ANHPI subgroup vs. Chinese.

	Heart failure	Ischemic heart disease	Stroke/transient ischemic attack
	HR (95% CIs)^d^	HR (95% CIs)^d^	HR (95% CIs)^d^
Chinese	1 (ref)	1 (ref)	1 (ref)
Japanese	1.01 (0.75, 1.37)	0.99 (0.71, 1.39)^c^	0.92 (0.63, 1.34)
Filipino	1.61 (1.21, 2.15)	1.15 (0.82, 1.61)^c^	0.92 (0.63, 1.35)
Hawaiian	1.43 (0.90, 2.28)	1.26 (0.75, 2.12)^c^	0.83 (0.43, 1.61)
Korean	1.42 (0.99, 2.05)	1.08 (0.71, 1.63)^c^	0.61 (0.35, 1.07)
Vietnamese	1.25 (0.88, 1.77)	1.19 (0.82, 1.73)^c^	0.83 (0.52, 1.32)
Indian or Pakistani	2.15 (1.34, 3.46)	1.76 (1.05, 2.96)^c^	1.13 (0.60, 2.11)
Other Southeast Asian^a^	2.47 (1.24, 4.91)	2.05 (0.94, 4.47)^c^	2.05 (0.88, 4.81)
Pacific Islander	2.52 (1.33, 4.77)	0.69 (0.25, 1.90)^c^	0.91 (0.37, 2.28)
Other Asian^b^	1.06 (0.72, 1.57)	0.93 (0.60, 1.44)^c^	1.20 (0.79, 1.84)

Abbreviations: ANHPI, Asian, Native Hawaiian, and Pacific Islander; CIs, Confidence intervals; HR, Hazard ratio; NHW, Non‐Hispanic White.

^a^ Other Southeast Asian: including Laotian, Hmong, Kampuchean, and Thai.

^b^ Other Asian: including Asian, NOS and Oriental, NOS.

^c^ Proportional assumption model does not meet; use flexible parameter survival model.

^d^ COX proportional hazard model, adjusting for diagnosis age, diagnosis year, sex, and registry.

Potential demographic (Table 4) and clinical (Table 5) CVD risk factors were evaluated among ANHPI and NHW (Tables S6 and S7) lung cancer survivors. For ANHPI lung cancer patients, males had a higher risk of heart failure and ischemic heart disease than females. The risk for heart failure increased with age at lung cancer diagnosis (*p* for trend = 0.01). Higher baseline CCI and lower education were associated with a higher hazard or risk of heart failure and ischemic heart disease. Higher income level was not associated with the risk of heart failure and ischemic heart disease among ANHPI lung cancer survivors. More advanced stage at lung cancer diagnosis was associated with a higher risk of heart failure. ANHPI lung cancer patients treated with radiation therapy or chemotherapy had a higher risk of heart failure or ischemic heart disease, but those who underwent surgery had a lower risk of these two diseases. ANHPI lung cancer patients who had more chemotherapy claims also had a higher risk of heart failure but without a dose–response relationship (*p* for trend = 0.50).

**TABLE 4:** Potential demographic risk factors for CVD among ANHPI Lung Cancer Survivors.

	Heart failure		Ischemic heart disease		Stroke/transient ischemic attack	
	*N*(case)/*N*(total)	HR (95% CIs)	*N*(case)/*N*(total)	HR (95% CIs)	*N*(case)/*N*(total)	HR (95% CIs)
Sex						
Female	227/1681	1 (ref)	191/1473	1 (ref)	169/1793	1 (ref)
Male	255/1667	1.38 (1.15, 1.65)	196/1275	1.40 (1.15, 1.71)	117/1780	0.81 (0.64, 1.03)
Age at diagnosis, year^a^						
66–70	101/801	1 (ref)	101/687	1 (ref)	65/822	1 (ref)
71–75	118/977	1.08 (0.83, 1.41)	124/783	1.15 (0.88, 1.50)	83/1024	1.03 (0.75, 1.43)
76–80	125/854	1.47 (1.12, 1.92)	82/693	0.92 (0.68, 1.23)	78/909	1.16 (0.83, 1.63)
81–85	97/497	2.44 (1.83, 3.25)	57/392	1.41 (1.01, 1.97)	44/548	1.30 (0.88, 1.92)
86+	41/219	2.93 (2.01, 4.28)	23/193	1.45 (0.91, 2.33)	16/270	1.15 (0.66, 2.02)
Charlson Comorbidity Index (CCI) at baseline^b^						
0	151/1426	1 (ref)	153/1240	1 (ref)	109/1449	1 (ref)
1	181/1130	1.55 (1.25, 1.93)	140/922	1.29 (1.02, 1.62)	106/1205	1.30 (0.99, 1.70)
2+	150/792	2.15 (1.70, 2.71)	94/586	1.52 (1.16, 1.98)	71/919	1.33 (0.98, 1.81)
Registry area^c^						
West	435/2980	1 (ref)	> 340/2461	1 (ref)	248/3187	1 (ref)
Northeast	29/219	0.93 (0.63, 1.37)	25/164	1.05 (0.69, 1,59)	27/228	1.60 (1.06, 2.41)
Midwest	^j^/47	1.25 (0.62, 2.53)	< 11/31^i^	1.30 (0.52, 3.06)	^j^/50	0.85 (0.27, 2.65)
South	^j^/102	0.69 (0.37, 1.30)	11/92	0.80 (0.44, 1.48)	^j^/108	1.04 (0.51, 2.11)
Urbanization^d^						
Urban	453/3202	1 (ref)	366/2625	1 (ref)	> 275/3410	1 (ref)
Rural	29/146	1.48 (1.01, 2.16)	21/123	1.30 (0.83, 2.02)	< 11/163 ^j^	0.67 (0.33, 1.35)
Education: proportion above college^e^ (Census tract)^f^						
0%–40%	93/488	1 (ref)	67/382	1 (ref)	35/528	1 (ref)
< 40%–60%	166/1140	0.71 (0.55, 0.92)	127/958	0.66 (0.49, 0.90)^h^	89/1231	1.03 (0.69, 1.53)
< 60%–80%	169/1214	0.67 (0.52, 0.87)	149/1004	0.76 (0.57, 1.03)^h^	110/1291	1.14 (0.78, 1.68)
< 80%–100%	54/501	0.54 (0.38, 0.76)	44/403	0.55 (0.38, 0.81)^h^	50/518	1.37 (0.89, 2.13)
Income: median income in census tract^g^						
≤ 40,000	114/703	1 (ref)	84/566	1 (ref)	63/763	1 (ref)
40,000–60,000	149/989	1.09 (0.84, 1.42)	113/823	1.02 (0.75, 1.38)	64/1058	0.66 (0.45, 0.95)
60,000–80,000	115/838	1.22 (0.89, 1.66)	101/715	1.21 (0.85, 1.72)	81/898	0.92 (0.62, 1.38)
> 80,000	104/813	1.46 (0.99, 2.15)	89/643	1.42 (0.92, 2.20)	76/849	0.94 (0.57, 1.53)

Abbreviations: ANHPI, Asian, Native Hawaiian, and Pacific Islander; CIs, Confidence intervals; HR, Hazard ratio.

^a^ Adjusting for sex, diagnosis year, CCI, urbanization, registry area, income census index, education census index, histology, and stage of lung cancer.

^b^ Adjusting for sex, registry area, urbanization, income census index, education census index, diagnosis age, diagnosis year, and tobacco use.

^c^ Adjusting for sex, urbanization, income census index, education census index, diagnosis age, diagnosis year.

^d^ Adjusting for sex, income census index, education census index, age at diagnosis year of diagnosis.

^e^ Including some college and at least 4 years of college.

^f^ Adjusting for sex, registry area, urbanization, diagnosis age, and diagnosis year.

^g^ Adjusting for sex, registry area, urbanization, diagnosis age, diagnosis year, education census index.

^h^ Proportional assumption model does not meet; use flexible parameter survival model.

^i^ Counts < 11 are not shown; Centers for Medicare & Medicaid Services (CMS) Cell Suppression Policy.

^j^ Hidden because counts < 11, Centers for Medicare & Medicaid Services (CMS) Cell Suppression Policy.

**TABLE 5:** Potential clinical risk factors for CVD among ANHPI lung cancer survivors.

	Heart failure		Ischemic heart disease		Stroke/transient ischemic attack	
	*N*(case)/*N*(total)	HR (95% CIs)	*N*(case)/*N*(total)	HR (95% CIs)	*N*(case)/*N*(total)	HR (95% CIs)
Tobacco use^a^						
No	404/2842	1 (ref)	325/2368	1 (ref)	250/3027	1 (ref)
Yes	78/506	1.09 (0.85, 1.41)	62/380	1.19 (0.90, 1.58)	36/546	0.91 (0.63, 1.32)
Histology^b^						
SCLC	20/175	1 (ref)	11/143	1 (ref)	11/190	1 (ref)
NSCLC	441/3024	0.70 (0.44, 1.10)	360/2476	1.04 (0.57, 1.91)	259/3209	0.61 (0.33, 1.12)
Unspecified	21/149	0.80 (0.43, 1.50)	16/129	1.30 (0.60, 2.84)	16/174	0.91 (0.42, 1.97)
Origin of primary Laterality^c^						
Right	272/1890	1 (ref)	209/1540	1 (ref)	157/2051	1 (ref)
Left	201/1377	1.01 (0.84, 1.21)	173/1134	1.13 (0.93, 1.39)	126/1438	1.16 (0.91, 1.46)
Stage^d^						
Localized	177/978	1 (ref)	148/764	1 (ref)	113/1063	1 (ref)
Regional	160/974	1.14 (0.92, 1.41)	132/791	1.10 (0.87, 1.39)	81/1025	0.89 (0.67, 1.18)
Distant	145/1396	1.38 (1.10, 1.74)	107/1193	1.09 (0.84, 1.41)	92/1485	1.26 (0.95, 1.67)
Radiation therapy^e^						
No	> 308/2209	1 (ref)	> 262/1785	1 (ref)	> 199/2345	1 (ref)
Yes	163/1107	1.46 (1.19, 1.79)^f^	114/936	1.21 (0.96, 1.53)^f^	76/1195	1.06 (0.80, 1.40)
Unknown	< 11/32^g^	2.27 (0.84, 6.17)^f^	< 11/27^g^	1.46 (0.46, 4.62)^f^	< 11/33^g^	2.36 (0.95, 5.81)
Chemotherapy^e^						
No/unknown	293/1783	1 (ref)	227/1433	1 (ref)	174/1936	1 (ref)
Yes	189/1565	1.13 (0.90, 1.41)	160/1315	1.30 (1.01, 1.66)	112/1637	1.11 (0.83, 1.49)
Surgery^e^						
No	271/2098	1 (ref)	196/1773	1 (ref)	147/2273	1 (ref)
Yes	210/1246	0.51 (0.40, 0.64)	191/971	0.57 (0.44, 0.75)	138/1295	0.78 (0.57, 1.06)
Number of Chemotherapy Claims (from Medicare)^e^						
Categorical variable						
*N* = 0	254/1671	1 (ref)	206/1349	1 (ref)	152/1813	1 (ref)
1 ≤ *N* ≤ 8	76/510	1.31 (1.00, 1.72)^f^	62/422	1.19 (0.88, 1.60)	40/539	1.22 (0.85, 1.77)
8 < *N* ≤ 14	43/343	1.33 (0.95, 1.88)^f^	38/290	1.19 (0.83, 1.72)	19/361	0.94 (0.57, 1.55)
14 < *N* ≤ 24	48/424	1.19 (0.85, 1.66)^f^	47/347	1.37 (0.97, 1.94)	37/441	1.51 (1.02, 2.25)
*N* > 24	61/400	1.42 (1.05, 1.94)^f^	34/340	0.91 (0.61, 1.34)	38/419	1.35 (0.91, 2.01)
*p* trend		0.50		0.72		0.50
Continuous variable						
Every 5 claims		1.02 (1.00, 1.05)		0.99 (0.96, 1.03)		1.02 (0.99, 1.05)
Number of immunotherapy claims (from Medicare)^e^						
Categorical variable						
*N* = 0	443/3084	1 (ref)	364/2534	1 (ref)	262/3306	1 (ref)
1 ≤ *N* ≤ 3	14/79	2.44 (1.40, 4.23)	^h^/67	1.04 (0.48, 2.22)	^h^/82	1.88 (0.94, 3.74)
3 < *N* ≤ 7	^h^/69	1.45 (0.76, 2.76)	^h^/55	1.03 (0.48, 2.23)	^h^/69	0.97 (0.40, 2.39)
7 < *N* ≤ 15	^h^/54	1.54 (0.81, 2.93)	^h^/44	0.65 (0.24, 1.77)	^h^/53	1.38 (0.56, 3.39)
*N* > 15	^h^/62	0.50 (0.20, 1.21)	^h^/48	0.49 (0.20, 1.19)	^h^/63	0.74 (0.27, 2.01)
*p* trend		0.17		0.04		0.17
Continuous variable						
Every 3 claims		0.96 (0.90, 1.02)		0.94 (0.86, 1.02)		0.96 (0.88, 1.04)

Abbreviations: ANHPI, Asian, Native Hawaiian, and Pacific Islander; CIs, Confidence Intervals; HR, Hazard Ratio.

^a^ Adjusting for sex, CCI, registry area, urbanization, income census index, education census index, diagnosis age, diagnosis year.

^b^ Adjusting for sex, CCI, registry area, urbanization, income census index, education census index, diagnosis age, and diagnosis year, tobacco use.

^c^ Adjusting for sex, CCI, diagnosis age, diagnosis year.

^d^ Adjusting for sex, registry area, urbanization, diagnosis year, and tobacco use.

^e^ Adjusting for sex, CCI, registry area, urbanization, income census index, education census index, diagnosis age, and diagnosis year, histology, and stage.

^f^ Proportional assumption model does not meet; use flexible parameter survival model.

^g^ Counts < 11 are not shown, Centers for Medicare and Medicaid Services (CMS) Cell Suppression Policy.

^h^ Hidden because counts < 11, Centers for Medicare & Medicaid Services (CMS) Cell Suppression Policy.

In the Fine‐Gray competing risk model (Tables S8 and S9), the results were similar to the results of the Cox proportional model, except that in the competing risk model, the risk of heart failure was lower in Filipino lung cancer patients than in NHW lung cancer patients, and the risk of heart failure in other Asian lung cancer patients and the risk of ischemic heart disease in Japanese lung cancer patients were not different from the risks in NHW lung cancer patients. When compared to Chinese lung cancer patients, there was no difference in the risk of heart failure in other Southeast Asian lung cancer patients, and the risk of stroke in Korean lung cancer patients was lower in the competing risk model.

## 4 Discussion

Previous studies also reported that Asian/Pacific Islander lung cancer survivors were less likely to have surgery than NHW lung cancer patients [22, 23]. One of these studies identified 61,961 lung cancer patients from SEER between 2004 and 2017 and reported that Asian/Pacific Islander lung cancer survivors received radiation therapy at a lower proportion but received chemotherapy at a similar proportion compared to NHW, which was different from our results [23]. The difference might be because ANHPI lung cancer survivors in our study are older and also more likely to be covered by Medicare, which increases the likelihood of receiving chemotherapy. Additionally, ANHPI lung cancer patients were more often diagnosed with adenocarcinoma and at an advanced stage compared to NHW lung cancer patients, which was consistent with previous studies [23, 24, 25, 26]. Furthermore, ANHPI lung cancer patients had a lower mortality rate than NHW lung cancer patients, similar to the findings from previous studies [26, 27, 28, 29].

A prospective cohort study between 1999 and 2000 in the United States reported that among individuals without prevalent CVD, 271,102 Asian American dialysis patients had a 28% decreased risk of combined non‐fatal and fatal myocardial infarction, which is a component of ischemic heart disease [30]. This study was consistent with our outcomes, but the study population was dialysis patients who received renal replacement therapy, rather than lung cancer patients. In the general population, ANHPIs have a lower prevalence of CVD [3, 31, 32], as well as CVD risk factors [31, 33] than the NHW population. Another study using the National Health Interview Survey reported that the Asian population had a lower odds of coronary heart disease than the NHW population [34]. In terms of the risk of stroke, we observed that there was no difference between ANHPI and NHW lung cancer survivors, which was inconsistent with previous studies that had suggested a slightly lower risk of stroke for ANHPI individuals compared with NHW individuals in the general population [31, 34].

Compared to NHW lung cancer patients, the lower risk of heart failure for Chinese and Japanese lung cancer patients may be due to a lower prevalence of obesity and smoking, or CVD‐related biomarkers (e.g., adipokine) among these subgroups [31, 35, 36]. When compared with the Chinese population, the higher risk of heart failure for Filipino and Indian/Pakistani lung cancer patients was consistent with previous studies not focused on cancer patients. A large cohort study including 1940 hypercholesterolemia patients reported that a variant in the prothrombin gene was strongly associated with the risk of CVD. This variant is more prevalent in India [37, 38]. Filipino and Indian/Pakistani individuals were also reported to have a higher prevalence of CVD risk factors (e.g., obesity, hyperlipidemia, diabetes, hypertension) in previous studies [39, 40, 41]. Higher CVD risks among the Filipino lung cancer patients may also be related to socioeconomic status [42].

A report on stroke in men and women mentioned that stroke was the third leading cause of death in females, compared with fifth in males in the United States [43]. This may partially explain why we observed a slightly higher risk of stroke in female lung cancer patients compared to male lung cancer patients. We also observed an increased risk of CVD conditions for higher baseline CCI. Some baseline comorbidities were associated with higher CVD risks, such as diabetes and renal disease [20]. For education and income, although previous studies suggested a protective effect of socioeconomic status on CVD [44, 45], our findings are supported by a study that reported education shows a stronger inverse association with obesity and diabetes, but not income [46]. We also observed higher CVD risks in lung cancer patients at advanced cancer stages. We excluded lung cancer patients diagnosed at an advanced stage and survived < 1 year; thus, the lung cancer patients at an advanced stage in our study who survived may have a higher chance of being diagnosed with CVD.

Previous studies have reported on radiation and chemotherapy cardiotoxicity [8, 47, 48, 49]. Surgery was associated with a lower incidence of CVD in our study, which may be explained by having a healthier baseline for the survivors who could undergo surgical resection [50]. Previous studies reported that pre‐existing CVD in lung cancer patients might reduce the likelihood of receiving chemotherapy by 47%, radiotherapy by 34%, and surgery by 44% [51, 52]. Since we excluded lung cancer survivors with prevalent CVD, our study may not be representative of the therapy distribution among all survivors of lung cancer.

We observed that ANHPI lung cancer patients were more likely to be diagnosed at an advanced stage than NHW lung cancer patients. We also found that distant stage was associated with a higher risk of heart failure in both ANHPI and NHW lung cancer patients. However, the risk of heart failure for ANHPI lung cancer patients was lower than that of NHW lung cancer patients. The lower risk of heart failure among ANHPI lung cancer patients may be due to differences in treatment patterns within this group. We found that ANHPI lung cancer patients were less likely to receive radiation therapy than NHW patients, and radiation therapy was associated with a higher risk of heart failure for both ANHPI and NHW lung cancer patients. Previous studies also found that Asian lung cancer patients were more likely to be diagnosed at a later stage than NHW lung cancer patients, and less likely to receive cancer‐related therapy [53, 54] but more likely to receive guideline‐concordant treatment [55]. Therefore, ANHPI lung cancer patients may receive less cardiotoxic cancer therapy and may experience more standard therapy after lung cancer diagnosis.

One major strength of this study is that we identified a large sample size of older ANHPI lung cancer patients, which was underrepresented in the United States, from a large dataset. To our knowledge, this is the first study to investigate the disparity of CVD risks for ANHPI lung cancer patients compared with NHW lung cancer patients, as well as comparisons among specific ANHPI subgroups. This is also the first study to investigate the CVD risks among ANHPI lung cancer subgroups and to test the heterogeneity of CVD risks among ANHPI subgroups after a lung cancer diagnosis. The SEER‐Medicare linked dataset allowed us to follow survivors from 2000 to 2019, which is long term in follow‐up time for lung cancer survivors. Additionally, the comparison of racial/ethnicity for CVD risks from K‐M curve and Cox proportional model was similar to the results from CIF curve and Fine‐Gray competing risk model, so the study results from different models were consistent in this study.

Because the 5‐year survival rate of lung cancer is only 22%, which is relatively low [9], about 75% of survivors in our study were followed up for < 5 years. We had limited statistical power to detect the CVD association among survivors with more than 5 years of follow‐up time, especially for some Asian subgroups. Furthermore, we excluded lung cancer survivors who were followed up for < 1 year to limit temporal ambiguity for lung cancer diagnosis and CVD incidence. Thus, we had fewer advanced‐stage patients and patients with a short survival time. Based on the result that ANHPI lung cancer patients were more likely to be diagnosed at an advanced stage, if CVD risks were associated with follow‐up time, we may have selection bias by excluding those who had 1 year or less of follow‐up time. Additionally, because we only matched on overall ANHPI lung cancer patients, the baseline distributions among ANHPI subgroups and NHW lung cancer patients may be unbalanced. Therefore, the comparison of marginal incidence between ANHPI subgroups and NHW may be confounded.

Another limitation may be caused by surveillance bias. ANHPI lung cancer patients and their subgroups may have a lower and heterogeneous Medicare enrollment rate compared with NHW lung cancer patients [56]. Therefore, lower risks of CVD in our results could be due to the disparities in health insurance enrollment and coverage. Moreover, we excluded lung cancer survivors who were enrolled in HMOs to ensure that we could capture all medical records of the lung cancer patients in the current study. A previous study indicated that cancer patients enrolled in HMOs were younger and diagnosed at an earlier stage [57]. Thus, the exclusion of HMO‐enrolled patients may result in an increased risk of CVDs for the participants in our study. We conducted a sensitivity analysis by checking the changes in HMO enrollment from 1999 to 2019 for each cancer registry region. For an area with a weighted change of more than 10% over time (California), we excluded participants from that area and conducted the analysis to assure that the inferences did not change. We presented the results including all registries because the sensitivity analysis did not show differences in our results. We also had limited information about behavior and treatment data. For example, we lacked information on cigarette consumption, which may be a confounder between potential CVD risk factors and CVD outcomes. Low sensitivity of radiation and chemotherapy data may also cause information bias [58]. We did identify lung cancer patients with tobacco use through the master beneficiary summary file (MBSF), but we expect this to be an underrepresentation of patients who smoked. Additionally, our HR estimates for the risk of CVD associated with other factors, such as baseline hypertension, dyslipidemia, and the family history of CVD, might also be biased due to these unmeasured confounders. Finally, although our overall ANHPI lung cancer patient group was large, some of the ANHPI subgroup patient numbers were small and limited our statistical power to detect associations.

## 5 Conclusion

In conclusion, we observed a statistically significant lower incidence of heart failure and ischemic heart disease among overall ANHPI patients with lung cancer, compared to NHW patients with lung cancer. Within ANHPI subgroups, Indian or Pakistani lung cancer patients experienced higher risks of these two CVD conditions compared to Chinese lung cancer patients. This implies that the racial/ethnic heterogeneity in CVD risks observed in the general population is mirrored in lung cancer survivors [9, 10]. Our study indicated the importance of focusing on the racial disparity issue among ANHPI subgroups when exploring relationships between lung cancer and CVD and in health care after the diagnosis of lung cancer. Further research is needed to investigate the reasons for the elevated risks of several CVDs among Pacific Islander, South Asian, and Southeast Asian lung cancer patients.

## Author Contributions

**Yancen Pan:** formal analysis (equal), investigation (equal), methodology (equal), software (equal), visualization (equal), writing – original draft (equal). **Chun‐Pin Esther Chang:** conceptualization (equal), data curation (equal), investigation (equal), methodology (equal), software (equal), validation (equal), writing – review and editing (equal). **Randa Tao:** writing – review and editing (equal). **Anees Daud:** writing – review and editing (equal). **Jincheng Shen:** writing – review and editing (equal). **Nathan D. Wong:** writing – review and editing (equal). **Roch A. Nianogo:** writing – review and editing (equal). **Jianyu Rao:** writing – review and editing (equal). **Thomas Varghese:** writing – review and editing (equal). **Zuo‐Feng Zhang:** writing – review and editing (equal). **Mia Hashibe:** conceptualization (equal), data curation (equal), funding acquisition (equal), investigation (equal), methodology (equal), project administration (equal), resources (equal), supervision (equal), writing – review and editing (equal).

## Ethics Statement

This is an observational study. We obtained approval for this study from the University of Utah Institutional Review Board.

## Consent

Under the IRB regulations, this study received approval for a waiver of informed consent.

## Conflicts of Interest

The authors declare no conflicts of interest.

## Supporting information


Appendix S1

## Data Availability

The datasets used to conduct this study are available upon approval of a research protocol from the National Cancer Institute. Instructions for obtaining these data are available at https://healthcaredelivery.cancer.gov/seermedicare/obtain/.
