# Supplementary material for: Cardiovascular Disease Risk Among Older Asian, Native Hawaiian, Pacific Islanders Lung Cancer Survivors
Source: Cancer Med. 2025 Feb 20;14(4):e70702. doi: 10.1002/cam4.70702 (PMC11840846; doi:10.1002/cam4.70702)

Supplementary Tables

|  | Heart Failure | | Ischemic Heart Disease | | | | Stroke/Transient Ischemic Attack | | |  |
| --- | --- | --- | --- | --- | --- | --- | --- | --- | --- | --- |
|  | Hazard Ratio (95% CIs) | | Hazard Ratio (95% CIs) | | | | Hazard Ratio (95% CIs) | | |  |
|  | Male | Female | Male | Female | | | Male | Female | |  |
| NHW |  |  | 1 (ref) | |  |  | | |  | |
| Overall ANHPI | 0.59 (0.45, 0.77) | 0.66 (0.52, 0.85) | 0.66 (0.46, 0.95) | 0.80 (0.60, 1.07) | | | 0.75 (0.52, 1.09) | 1.11 (0.83, 1.49) | |  |
| Chinese | 0.51 (0.33, 0.78) | 0.55 (0.34, 0.88) | 0.62 (0.34, 1.13) | 0.66 (0.38, 1.14) | | | 0.84 (0.46, 1.51) | 1.17 (0.72, 1.90) | |  |
| Japanese | 0.51 (0.27, 0.97) | 0.44 (0.26, 0.74) | 0.72 (0.33, 1.56) | 0.57 (0.32, 0.99) | | | 0.45 (0.17, 1.18) | 1.35 (0.73, 2.52) | |  |
| Filipino | 0.81 (0.48, 1.37) | 0.86 (0.54, 1.36) | 1.12 (0.56, 2.26) | 0.75 (0.42, 1.34) | | | 1.13 (0.54, 2.35) | 0.86 (0.46, 1.61) | |  |
| Hawaiian | 0.42 (0.15, 1.18) | 1.38 (0.53, 3.59) | 0.93 (0.29, 3.00) | 1.84 (0.57, 5.96) | | | 0.98 (0.21, 4.68) | 1.12 (0.29, 4.30) | |  |
| Korean | 0.64 (0.35, 1.19) | 0.90 (0.42, 1.90) | 0.86 (0.36, 2.06) | 0.89 (0.39, 2.05) | | | 0.68 (0.25, 1.83) | 0.63 (0.26, 1.55) | |  |
| Vietnamese | 0.43 (0.24, 0.76) | 0.88 (0.49, 1.59) | 0.42 (0.18, 0.99) | 1.02 (0.50, 2.09) | | | 0.52 (0.21, 1.30) | 0.94 (0.47, 1.88) | |  |
| Indian or Pakistani | 0.92 (0.40, 2.08) | 0.57 (0.23, 1.38) | 1.02 (0.29, 3.54) | 1.72 (0.54, 5.54) | | | 0.84 (0.29, 2.42) | 0.83 (0.18, 3.71) | |  |
| Other Southeast Asian ^a^ | 0.32 (0.04, 2.77) | 2.16 (0.62, 7.53) | 0.65 (0.06, 6.70) | 3.70 (0.84, 16.38) | | | 0.95 (0.16, 5.75) | 1.19 (0.27, 5.16) | |  |
| Pacific Islander | 2.42 (0.20, 29.68) | 0.74 (0.22, 2.51) | - ^c^ | 0.58 (0.14, 2.37) | | | - ^c^ | 2.89 (0.65, 12.94) | |  |
| Other Asian ^b^ | 0.70 (0.31, 1.56) | 0.42 (0.19, 0.93) | 0.27 (0.07, 1.00) | 0.55 (0.22, 1.36) | | | 0.92 (0.35, 2.42) | 1.80 (0.89, 3.62) | |  |

**Supplementary Table 1. Risk of CVDs among Lung Cancer Survivors, ANHPI and Subgroup V.S. NHW, by sex.**

a. Other Southeast Asian: including Laotian, Hmong, Kampuchean and Thai.

b. Other Asian: including Asian, NOS and Oriental, NOS.

c. No result due to 0 event.

d. ANHPI: Asian, Native Hawaiian, and Pacific Islander; NHW: Non-Hispanic White

e. HR: Hazard Ratio; CIs: Confidence Intervals.

f. COX proportional hazard model, adjusting for matched pair and registry.

**Supplementary Table 2. The Risk of CVDs among Lung Cancer Survivors, ANHPI and Subgroup V.S. NHW, by Years since Diagnosis**

|  | Heart Failure | | Ischemic Heart Disease | Stroke/Transient Ischemic Attack | | | | |
| --- | --- | --- | --- | --- | --- | --- | --- | --- |
|  | 1-5 years | > 5 years | 1-5 years | | >5 years | 1-5 years | > 5 years | |
|  | HR (95% CIs) | HR (95% CIs) | HR (95% CIs) | | HR (95% CIs) | HR (95% CIs) | HR (95% CIs) | |
| NHW | 1 (ref) | | | | | | |  |
| Overall ANHPI | 0.63 (0.52, 0.77) | 0.63 (0.37, 1.07) | 0.74 (0.58, 0.93) | | 0.87 (0.43, 1.76) | 1.14 (0.94, 1.38) ^d^ | 1.70 (0.86, 3.36) | |
| Chinese | 0.56 (0.40, 0.78) | 0.28 (0.10, 0.82) | 0.62 (0.41, 0.94) | | 2.13 (0.31, 14.70) | 1.05 (0.71, 1.55) | 1.38 (0.32, 5.95) | |
| Japanese | 0.47 (0.31, 0.72) | 0.54 (0.18, 1.64) | 0.76 (0.47, 1.21) | | 0.09 (0.01, 0.84) | 0.87 (0.49, 1.53) | 1.45 (0.43, 4.95) | |
| Filipino | 0.79 (0.55, 1.15) | 1.12 (0.43, 2.93) | 0.85 (0.53, 1.34) | | 0.91 (0.18, 4.67) | 1.02 (0.63, 1.66) | 1.26 (0.17, 9.67) | |
| Hawaiian | 0.57 (0.26, 1.22) | 7.11 (0.68, 74.44) | 0.99 (0.41, 2.42) | | - ^c^ | 1.22 (0.42, 3.56) | 0.38 (0.03, 4.86) | |
| Korean | 0.68 (0.41, 1.13) | 1.32 (0.31, 5.63) | 0.64 (0.33, 1.26) | | 3.79 (0.60, 24.01) | 0.65 (0.32, 1.30) | 0.61 (0.06, 6.06) | |
| Vietnamese | 0.62 (0.40, 0.96) | 0.48 (0.14, 1.62) | 0.68 (0.38, 1.23) | | 1.16 (0.27, 5.02) | 0.59 (0.32, 1.08) | 5.49 (0.87, 34.51) | |
| Indian or Pakistani | 0.71 (0.37, 1.35) | 0.54 (0.10, 2.82) | 1.33 (0.55, 3.23) | | 2.28 (0.20, 26.63) | 0.98 (0.37, 2.59) | 1.81 (0.23, 14.53) | |
| Other Southeast Asian ^a^ | 1.26 (0.47, 3.38) | - ^c^ | 2.33 (0.67, 8.06) | | - ^c^ | 1.00 (0.32, 3.07) | - ^c^ | |
| Pacific Islander | 1.16 (0.39, 3.45) | - ^c^ | 0.33 (0.07, 1.60) | | 0.56 (0.05, 6.97) | 0.88 (0.21, 3.67) | 2.31 (0.23, 23.59) | |
| Other Asian ^b^ | 0.53 (0.29, 0.98) | 0.40 (0.08, 1.87) | 0.48 (0.23, 1.02) | | - ^c^ | 1.17 (0.64, 2.15) | 5.69 (0.83, 38.77) | |
| a. Other Southeast Asian: including Laotian, Hmong, Kampuchean and Thai.  b. Other Asian: including Asian, NOS and Oriental, NOS.  c. No result due to 0 event.  d. Proportional assumption model does not meet, use flexible parameter survival model.  e. ANHPI: Asian, Native Hawaiian, and Pacific Islander; NHW: Non-Hispanic White  f. HR: Hazard Ratio; CIs: Confidence Intervals.  g. COX proportional hazard model, adjusting for matched pair and registry. | | | | | | | | |

**Supplementary Table 3. The Risk of CVDs among Lung Cancer Survivors, Sub-ANHPI V.S. Chinese lung Cancer Patients, by Years since Diagnosis**

|  | Heart Failure | | Ischemic Heart Disease | | Stroke/Transient Ischemic Attack | | | |
| --- | --- | --- | --- | --- | --- | --- | --- | --- |
|  | 1-5 years | > 5 years | 1-5 years | >5 years | | 1-5 years | > 5 years | |
|  | HR (95% CIs) | HR (95% CIs) | HR (95% CIs) | HR (95% CIs) | | HR (95% CIs) | HR (95% CIs) | |
| Chinese | 1 (ref) | | | | | | |  |
| Japanese | 1.24 (0.86, 1.77) ^d^ | 0.74 (0.42, 1.29) | 1.05 (0.72, 1.54) | 0.87 (0.43, 1.79) | | 0.83 (0.54, 1.29) | 1.22 (0.56, 2.70) | |
| Filipino | 1.64 (1.17, 2.31) ^d^ | 1.53 (0.89, 2.62) | 1.08 (0.74, 1.57) | 1.62 (0.78, 3.39) | | 0.99 (0.66, 1.49) | 0.51 (0.17, 1.59) | |
| Hawaiian | 1.39 (0.78, 2.45) ^d^ | 1.81 (0.80, 4.07) | 1.24 (0.68, 2.25) | 1.21 (0.39, 3.73) | | 0.81 (0.39, 1.67) | 1.02 (0.21,5.01) | |
| Korean | 1.51 (1.01, 2.27) ^d^ | 1.08 (0.47, 2.52) | 0.93 (0.58, 1.49) | 1.97 (0.83, 4.69) | | 0.61 (0.33, 1.11) | 0.52 (0.11, 2.36) | |
| Vietnamese | 1.44 (0.97, 2.14) ^d^ | 0.81 (0.39, 1.68) | 1.02 (0.66, 1.58) | 2.10 (0.99, 4.45) | | 0.78 (0.45, 1.33) | 1.04 (0.40, 2.72) | |
| Indian or Pakistani | 2.03 (1.14, 3.61) ^d^ | 2.34 (1.00, 5.47) | 1.67 (0.93, 3.02) | 1.44 (0.43, 4.87) | | 1.02 (0.49, 2.14) | 1.72 (0.50, 5.88) | |
| Other Southeast Asian ^a^ | 2.88 (1.43, 5.77) ^d^ | - ^c^ | 2.20 (1.01, 4.83) | - ^c^ | | 1.81 (0.71, 4.57) | 5.36 (0.60, 48.38) | |
| Pacific Islander | 3.69 (1.87, 7.30) ^d^ | 0.69 (0.09, 5.21) | 0.68 (0.21, 2.18) | 0.87 (0.11, 6.77) | | 0.71 (0.22, 2.28) | 1.60 (0.35, 7.41) | |
| Other Asian ^b^ | 1.01 (0.63, 1.63) ^d^ | 1.24 (0.60, 2.55) | 0.60 (0.33, 1.08) | 2.26 (1.07, 4.76) | | 1.13 (0.69, 1.84) | 1.82 (0.75, 4.41) | |
| a. Other Southeast Asian: including Laotian, Hmong, Kampuchean and Thai.  b. Other Asian: including Asian, NOS and Oriental, NOS.  c. No result for power issue.  d. Proportional assumption model does not meet, use flexible parameter survival model.  e. ANHPI: Asian, Native Hawaiian, and Pacific Islander; NHW: Non-Hispanic White  f. HR: Hazard Ratio; CIs: Confidence Intervals.  g. COX proportional hazard model, adjusting for matching factors and registry. | | | | | | | |  |

**Supplementary Table 4. The Risk of CVDs among Lung Cancer Survivors, ANHPI and Subgroup V.S. NHW, Multivariable Adjusted Models**

|  | Heart Failure | | Ischemic Heart Disease | | Stroke/Transient Ischemic Attack | | | |
| --- | --- | --- | --- | --- | --- | --- | --- | --- |
|  | Adjusted Model 1 ^a^ | Adjusted Model 2 ^b^ | Adjusted Model 1 | Adjusted Model 2 | | Adjusted Model 1 | Adjusted Model 2 | |
|  | HR (95% CIs) | HR (95% CIs) | HR (95% CIs) | HR (95% CIs) | | HR (95% CIs) | HR (95% CIs) | |
| NHW | 1 (ref) | | | | | | |  |
| Overall ANHPI | 0.67 (0.57, 0.79) | 0.70 (0.59, 0.82) | 0.83 (0.68, 1.02) | 0.84 (0.68, 1.03) | | 1.08 (0.91,1.28) ^e^ | 1.07 (0.90, 1.28) ^e^ | |
| Chinese | 0.54 (0.40, 0.72) | 0.56 (0.41, 0.75) | 0.79 (0.55, 1.15) | 0.80 (0.55, 1.15) | | 1.22 (0.87, 1.71) | 1.25 (0.89, 1.75) | |
| Japanese | 0.58 (0.41, 0.81) | 0.59 (0.42, 0.84) | 0.71 (0.47, 1.05) | 0.71 (0.47, 1.05) | | 1.09 (0.70, 1.69) | 1.11 (0.72, 1.73) | |
| Filipino | 0.93 (0.66, 1.31) | 0.97 (0.69, 1.37) | 0.95 (0.62, 1.45) | 0.95 (0.62, 1.47) | | 1.10 (0.69, 1.74) | 1.10 (0.69, 1.76) | |
| Hawaiian | 1.21 (0.68, 2.17) | 1.22 (0.68, 2.18) | 1.55 (0.75, 3.17) | 1.52 (0.74, 3.13) | | 1.22 (0.51, 2.95) | 1.22 (0.51, 2.95) | |
| Korean | 0.71 (0.44, 1.14) | 0.74 (0.46, 1.21) | 0.89 (0.49, 1.59) | 0.91 (0.50, 1.63) | | 0.68 (0.35, 1.31) | 0.70 (0.36, 1.35) | |
| Vietnamese | 0.52 (0.34, 0.79) | 0.55 (0.36, 0.83) | 0.64 (0.37, 1.11) | 0.63 (0.36, 1.09) | | 0.90 (0.53, 1.55) | 0.92 (0.53, 1.58) | |
| Indian or Pakistani | 0.83 (0.46, 1.52) | 0.87 (0.48, 1.59) | 1.83 (0.80, 4.19) | 1.85 (0.81, 4.24) | | 0.93 (0.40, 2.20) | 0.93 (0.39, 2.19) | |
| Other Southeast Asian ^c^ | 1.07 (0.40, 2.87) | 1.15 (0.43, 3.08) | 1.31 (0.40, 4.30) | 1.33 (0.40, 4.37) | | 1.16 (0.38, 3.53) | 1.20 (0.39, 3.69) | |
| Pacific Islander | 0.89 (0.31, 2.52) | 0.95 (0.33, 2.68) | 0.45 (0.12, 1.70) | 0.46 (0.12, 1.72) | | 0.94 (0.29, 3.00) | 0.97 (0.30, 3.10) | |
| Other Asian ^d^ | 0.59 (0.33, 1.03) | 0.62 (0.35, 1.09) | 0.48 (0.23, 1.00) | 0.49 (0.23, 1.02) | | 1.36 (0.78, 2.38) | 1.39 (0.79, 2.45) | |
| a. Adjusted model 1: adjust for registry area, matched pairs, urbanization, education, income, CCI.  b. Adjusted model 2: adjust for registry area, matched pairs, urbanization, education, income, CCI, smoking, alcohol, obesity.  c. Other Southeast Asian: including Laotian, Hmong, Kampuchean and Thai.  d. Other Asian: including Asian, NOS and Oriental, NOS.  e. Proportional assumption model does not meet, use flexible parameter survival model.  f. ANHPI: Asian, Native Hawaiian, and Pacific Islander; NHW: Non-Hispanic White  g. HR: Hazard Ratio; CIs: Confidence Intervals.  h. COX proportional hazard model, adjusting for matched pair and registry. | | | | | | | | |

**Supplementary Table 5. The Risk of CVDs among Lung Cancer Survivors, ANHPI Subgroup V.S. Chinese, Multivariable Adjusted Models**

|  | Heart Failure | | Ischemic Heart Disease | | Stroke/Transient Ischemic Attack | | |
| --- | --- | --- | --- | --- | --- | --- | --- |
|  | Adjusted Model 1 ^a^ | Adjusted Model 2 ^b^ | Adjusted Model 1 | Adjusted Model 2 | | Adjusted Model 1 | Adjusted Model 2 |
|  | HR (95% CIs) | HR (95% CIs) | HR (95% CIs) | HR (95% CIs) | | HR (95% CIs) | HR (95% CIs) |
| Chinese | 1 (reference) | | | | | | |
| Japanese | 0.96 (0.72, 1.28) | 1.03 (0.76, 1.41) ^e^ | 0.98 (0.70, 1.36) ^e^ | 0.95 (0.68, 1.33) ^e^ | | 0.92 (0.65, 1.30) | 0.92 (0.65, 1.30) |
| Filipino | 1.44 (1.08, 1.90) | 1.51 (1.13, 2.03) ^e^ | 1.10 (0.78, 1.54) ^e^ | 1.10 (0.79, 1.55) ^e^ | | 0.90 (0.62, 1.32) | 0.89 (0.61, 1.31) |
| Hawaiian | 1.34 (0.86, 2.09) | 1.37 (0.85, 2.21) ^e^ | 1.18 (0.70, 2.00) ^e^ | 1.13 (0.67, 1.93) ^e^ | | 0.95 (0.51, 1.78) | 0.93 (0.50, 1.76) |
| Korean | 1.33 (0.93, 1.90) | 1.40 (0.97, 2.02) ^e^ | 1.05 (0.69, 1.59) ^e^ | 1.06 (0.70, 1.60) ^e^ | | 0.54 (0.31, 0.95) | 0.55 (0.32, 0.96) |
| Vietnamese | 1.00 (0.71, 1.40) | 1.08 (0.76, 1.55) ^e^ | 1.08 (0.74, 1.58) ^e^ | 1.08 (0.74, 1.57) ^e^ | | 0.78 (0.50, 1.24) | 0.78 (0.50, 1.24) |
| Indian or Pakistani | 1.93 (1.22, 3.08) | 2.07 (1.28, 3.36) ^e^ | 1.75 (1.04, 2.95) ^e^ | 1.75 (1.04, 2.95) ^e^ | | 1.10 (0.59, 2.04) | 1.11 (0.60, 2.05) |
| Other Southeast Asian ^c^ | 2.34 (1.18, 4.64) | 2.58 (1.29, 5.16) ^e^ | 2.07 (0.95, 4.51) ^e^ | 2.02 (0.92, 4.40) ^e^ | | 1.88 (0.81, 4.34) | 1.93 (0.83, 4.45) |
| Pacific Islander | 2.08 (1.10, 3.91) | 2.37 (1.23, 4.57) ^e^ | 0.69 (0.25, 1.90) ^e^ | 0.68 (0.25, 1.87) ^e^ | | 0.84 (0.34, 2.08) | 0.82 (0.33, 2.05) |
| Other Asian ^d^ | 0.99 (0.67, 1.47) | 1.06 (0.71, 1.57) ^e^ | 0.92 (0.59, 1.43) ^e^ | 0.91 (0.59, 1.42) ^e^ | | 1.08 (0.71, 1.64) | 1.07 (0.70, 1.63) |
| a. Adjusted model 1: adjust for registry area, matched pairs, urbanization, education, income, CCI.  b. Adjusted model 2: adjust for registry area, matched pairs, urbanization, education, income, CCI, smoking, alcohol, obesity.  c. Other Southeast Asian: including Laotian, Hmong, Kampuchean and Thai.  d. Other Asian: including Asian, NOS and Oriental, NOS.  e. Proportional assumption model does not meet, use flexible parameter survival model.  f. ANHPI: Asian, Native Hawaiian, and Pacific Islander; NHW: Non-Hispanic White  g. HR: Hazard Ratio; CIs: Confidence Intervals.  h. COX proportional hazard model, adjusting for matched pair and registry. | | | | | | | |

| **Supplementary Table 6. Potential Demographic Risk Factors for CVD among NHW Lung Cancer Survivors.** | | | | | | | | | | | |  |
| --- | --- | --- | --- | --- | --- | --- | --- | --- | --- | --- | --- | --- |
|  | Heart Failure | | Ischemic Heart Disease | | | | Stroke/  Transient Ischemic Attack | |  |  |  |  |
|  | N (case)/  N (total) | HR  (95% CIs) | N (case)/  N (total) | | HR  (95% CIs) | | N (case)/  N (total) | HR  (95% CIs) |  |  |  |  |
| Sex |  |  | |  | |  | | | | |  | |
| female | 929/4503 | 1 (ref) | 619/3804 | | 1 (ref) | | 453/5227 | 1 (ref) |  |  |  |  |
| male | 878/4619 | 1.30 (1.18, 1.42) | 589/2962 | | 1.49 (1.33, 1.67) ^j^ | | 367/5271 | 0.95 (0.83, 1.09) |  |  |  |  |
| Age at diagnosis, year ^a^ | | | | | | | | | |  |  |  |
| 66-70 | 373/2228 | 1 (ref) | 302/1712 | | 1 (ref) | | 173/2429 | 1 (ref) |  |  |  |  |
| 71-75 | 494/2659 | 1.22 (1.07, 1.40) | 350/1960 | | 1.03 (0.88, 1.20) | | 243/3023 | 1.22 (1.01, 1.49) |  |  |  |  |
| 76-80 | 506/2303 | 1.67 (1.46, 1.92) | 293/1646 | | 1.21 (1.03, 1.43) | | 206/2663 | 1.36 (1.11, 1.67) |  |  |  |  |
| 81-85 | 309/1326 | 2.13 (1.83, 2.49) | 180/968 | | 1.56 (1.29, 1.88) | | 144/1619 | 1.83 (1.46, 2.29) |  |  |  |  |
| 86+ | 125/606 | 2.22 (1.80, 2.74) | 83/480 | | 1.67 (1.30, 2.15) | | 54/764 | 1.80 (1.32, 2.47) |  |  |  |  |
| Charlson Comorbidity Index (CCI) at baseline ^c^ | | | | | | | | | |  |  |  |
| 0 | 571/3635 | 1 (ref) | 441/2936 | | 1 (ref) | | 305/3788 | 1 (ref) |  |  |  |  |
| 1 | 691/3307 | 1.52 (1.35, 1.70) | 477/2501 | | 1.43 (1.26, 1.63) | | 284/3747 | 1.15 (0.98, 1.36) |  |  |  |  |
| 2+ | 545/2180 | 2.11 (1.87, 2.39) | 290/1329 | | 1.92 (1.64, 2.24) | | 231/2963 | 1.40 (1.17, 1.67) |  |  |  |  |
| Registry area ^d^ | | | | | | | | | |  |  |  |
| West | 587/3140 | 1 (ref) | 394/2454 | | 1 (ref) | | 258/3563 | 1 (ref) |  |  |  |  |
| Northeast | 438/2095 | 1.04 (0.91, 1.19) | 298/1475 | | 1.22 (1.03, 1.44) | | 232/2407 | 1.37 (1.13, 1.67) |  |  |  |  |
| Midwest | 248/1202 | 1.24 (1.06, 1.44) | 168/868 | | 1.38 (1.15, 1.67) | | 110/1436 | 1.34 (1.07, 1.69) |  |  |  |  |
| South | 534/2685 | 1.08 (0.95, 1.23) | 348/1969 | | 1.12 (0.95, 1.31) | | 220/3092 | 1.23 (1.01, 1.50) |  |  |  |  |
| Urbanization ^e^ | | | | | | | | | |  |  |  |
| Urban | 1471/7328 | 1 (ref) | 980/5419 | | 1 (ref) | | 681/8414 | 1 (ref) |  |  |  |  |
| Rural | 336/1792 | 0.86 (0.76, 0.98) | 228/1345 | | 0.86 (0.74, 1.01) | | 139/2082 | 0.94 (0.77, 1.15) |  |  |  |  |
| Education: proportion above college ^i^ (Census tract) ^f^ | | | | | | | | |  |  |  |  |
| 0%-40% | 369/1797 | 1 (ref) | 237/1301 | | 1 (ref) | | 127/2112 | 1 (ref) |  |  |  |  |
| < 40%-60% | 652/3186 | 0.95 (0.83, 1.09) | 440/2330 | | 0.98 (0.83, 1.16) ^j^ | | 287/3735 | 1.30 (1.05, 1.63) |  |  |  |  |
| < 60%-80% | 601/2937 | 0.87 (0.75, 1.00) | 389/2208 | | 0.89 (0.74, 1.07) ^j^ | | 285/3346 | 1.35 (1.07, 1.70) |  |  |  |  |
| < 80%-100% | 182/1192 | 0.60 (0.50, 0.73) | 140/919 | | 0.73 (0.58, 0.92) ^j^ | | 121/1294 | 1.42 (1.07, 1.87) |  |  |  |  |
| Income: median income in census tract ^g^ | | | | | | | | |  |  |  |  |
| ≤40,000 | 515/2486 | 1 (ref) | 355/1834 | | 1 (ref) | | 210/2914 | 1 (ref) |  |  |  |  |
| 40,000-60,000 | 621/3051 | 1.02 (0.89, 1.17) | 403/2282 | | 0.87 (0.74, 1.03) ^j^ | | 264/3552 | 0.91 (0.74, 1.13) |  |  |  |  |
| 60,000-80,000 | 373/1818 | 1.04 (0.87, 1.24) | 241/1350 | | 0.86 (0.69, 1.07) ^j^ | | 178/2085 | 0.91 (0.69, 1.19) |  |  |  |  |
| >80,000 | 295/1756 | 0.93 (0.74, 1.16) | 207/1291 | | 0.78 (0.60, 1.03) ^j^ | | 168/1935 | 0.86 (0.62, 1.19) |  |  |  |  |
| Tobacco use ^h^ | | | | | | | | |  |  |  |  |
| No | 1230/6145 | 1(ref) | 883/4743 | | 1(ref) | | 588/7054 | 1(ref) |  |  |  |  |
| Yes | 577/2977 | 1.14 (1.02, 1.26) ^j^ | 325/2023 | | 1.02 (0.90, 1.17) | | 232/3444 | 1.04 (0.89, 1.22) |  |  |  |  |

a. adjusting for sex, diagnosis year, CCI, urbanization, registry area, income census index, education census index, histology and stage of lung cancer.

b. adjusting for sex, CCI, urbanization, registry area, income census index, education census index, diagnosis age.

c. adjusting for sex, registry area, urbanization, income census index, education census index, diagnosis age, diagnosis year, tobacco use.

d. adjusting for sex, urbanization, income census index, education census index, diagnosis age, diagnosis year.

e. adjusting for sex, income census index, education census index, diagnosis age, diagnosis year.

f. adjusting for sex, registry area, urbanization, diagnosis age, diagnosis year.

g. adjusting for sex, registry area, urbanization, diagnosis age, diagnosis year, education census index.

h. adjusting for sex, CCI, registry area, urbanization, income census index, education census index, diagnosis age, diagnosis year.

i. Including some college and at least 4 years of college.

j. Proportional assumption model does not meet, use flexible parameter survival model.

k. NHW: Non-Hispanic White

l. HR: Hazard Ratio; CIs: Confidence Intervals

| **Supplementary Table 7. Potential Clinical Risk Factors for CVD among NHW Lung Cancer Survivors.** | | | | | | | | | | | | | | | | | |  |
| --- | --- | --- | --- | --- | --- | --- | --- | --- | --- | --- | --- | --- | --- | --- | --- | --- | --- | --- |
|  | Heart Failure | | | | | | Ischemic Heart Disease | |  | | | | Stroke/  Transient Ischemic Attack | | | | | |
|  | N (case)/  N (total) | | | HR  (95% CIs) | | | N (case)/  N (total) | HR  (95% CIs) |  | | | | N (case)/  N (total) | HR  (95% CIs) | | | | |
| Histology ^a^ | | | | | | | | | | | | | | | | | |  |
| SCLC | 95/742 | | | 1(ref) | | | 59/562 | 1(ref) | | 48/869 | | | | 1(ref) | | | |  |
| NSCLC | 1609/7865 | | | 0.79 (0.64, 0.98) | | | 1079/5793 | 0.92 (0.70, 1.20) | | 735/8971 | | | | 0.70 (0.52, 0.94) ^e^ | | | | |
| Unspecified | 103/515 | | | 0.94 (0.71, 1.25) | | | 70/411 | 1.03 (0.73, 1.47) | | 37/658 | | | | 0.63 (0.41, 0.98) ^e^ | | | | |
| Origin of primary Laterality ^b^ | | | | | | | | | | | | | | | | | |  |
| Right | 1014/5251 | | | 1 (ref) | | | 716/3896 | 1 (ref) | | | 470/6045 | | | | 1 (ref) | | |  |
| Left | 757/3671 | | | 1.04 (0.95, 1.15) | | | 467/2709 | 0.92 (0.82, 1.03) | | | 324/4220 | | | 0.98 (0.85, 1.13) | | | | |
| Stage ^c^ | | | | | | | | | | | | | | | | | |  |
| Localized | 852/3370 | | | | 1 (ref) | | 554/2411 | 1 (ref) | | | 357/3940 | | | | 1 (ref) | | |  |
| Regional | 574/3009 | | | | 0.97 (0.87, 1.08) | | 417/2176 | 1.04 (0.91, 1.18) | | | 272/3461 | | | 1.15 (0.98, 1.35) | | | | |
| Distant | 381/2743 | | | | 1.35 (1.20, 1.53) | | 237/2179 | 1.09 (0.93, 1.27) | | | 191/3097 | | | 1.76 (1.47, 2.11) | | | | |
| Radiation therapy ^d^ | | | | | | | | | | | | | | | | | |  |
| No | 1105/5469 | | | | 1 (ref) | | 758/4005 | 1 (ref) | | 552/6204 | | | | | | | 1 (ref) |  |
| Yes | 677/3508 | | | 1.47 (1.33, 1.63) ^e^ | | | 427/2660 | 1.23 (1.08, 1.39) |  | | | | 255/4125 | 1.05 (0.89, 1.23) | | | | |
| Unknown | 25/145 | | | 0.92 (0.62, 1.38) | | | 23/101 | 1.26 (0.83, 1.93) |  | | | | 13/169 | 1.03 (0.59, 1.79) | | | | |
| Chemotherapy ^d^ | | | | | | | | | | | | | | | | | |  |
| No/Unknown | 1213/5400 | 1 (ref) | | | | | 808/3959 | 1 (ref) | | 542/6328 | | | | | | | 1 (ref) |  |
| Yes | 594/3722 | 1.26 (1.12, 1.42) | | | | | 400/2807 | 1.14 (0.98, 1.32) |  | | | | 278/4170 | 1.23 (1.03, 1.47) | | | | |
| Surgery ^d^ | | | | | | | | | | | | | | | | | |  |
| No | 886/5261 | 1 (ref) | | | | | 584/4075 | 1 (ref) | | 372/6212 | | | | | | | 1 (ref) |  |
| Yes | 912/3813 | 0.65 (0.58, 0.72) | | | | | 616/2651 | 0.67 (0.58, 0.77) |  | | | | 448/4232 | 0.83 (0.70, 0.99) | | | | |
| Number of Chemotherapy Claims (from Medicare) ^d^ | | | | | | | | | | | | | | | | | | |
| Categorical variable |  | | |  | | |  |  |  | | | |  |  | | | | |
| N=0 | 1029/4541 | | | | | 1 (ref) | 685/3355 | 1 (ref) | | | | 463/5361 | | | 1 (ref) | | | |
| 1≤N≤8 | 218/1195 | | | | | 1.11 (0.96, 1.30) | 155/855 | 1.16 (0.96, 1.39) |  | | | | 117/1393 | 1.31 (1.05, 1.62) | | | | |
| 8<N≤14 | 1777/998 | | | | | 1.36 (1.15, 1.61) | 113/720 | 1.19 (0.97, 1.47) |  | | | | 78/1135 | 1.28 (0.99, 1.65) | | | | |
| 14<N≤24 | 192/1207 | | | | | 1.31 (1.11, 1.55) | 119/934 | 1.09 (0.89, 1.35) |  | | | | 72/1342 | 0.99 (0.76, 1.29) | | | | |
| N>24 | 191/1181 | | | | | 1.21 (1.02, 1.43) | 136/902 | 1.15 (0.94, 1.42) |  | | | | 90/1267 | 1.16 (0.90, 1.50) | | | | |
| P trend |  | | | 1.00 | | |  | 0.50 |  | | | |  | 0.17 | | | | |
| Continuous variable |  | | |  | | |  |  |  | | | |  |  | | | | |
| Every 5 Claims |  | | | 1.00 (0.99, 1.02) | | |  | 0.99 (0.98, 1.01) |  | | | |  | 1.00 (0.98, 1.02) | | | | |
| Number of Immunotherapy Claims (from Medicare) ^d^ | | | | | | | | | | | | | | | | | | |
| Categorical variable |  | | |  | | |  |  |  | | | |  |  | | | | |
| N=0 | 1716/8543 | | 1 (ref) | | | | 1142/6342 | 1 (ref) | | | | >770/9856 | | | | 1 (ref) | | |
| 1≤N≤3 | 24/152 | | 1.14 (0.76, 1.72) | | | | 19/115 | 1.30 (0.82, 2.05) |  | | | | 16/166 | 1.57 (0.95, 2.60) | | | | |
| 3<N≤7 | 24/136 | | 1.20 (0.80, 1.80) | | | | 14/99 | 1.22 (0.72, 2.08) |  | | | | 12/163 | 1.34 (0.75, 2.38) | | | | |
| 7<N≤15 | 24/139 | | 1.32 (0.88, 2.00) | | | | 16/99 | 1.52 (0.92, 2.51) |  | | | | <11/154 ^i^ | 0.82 (0.39, 1.74) | | | | |
| N>15 | 19/152 | | 0.55 (0.35, 0.88) | | | | 17/111 | 0.80 (0.49, 1.30) |  | | | | 11/159 | 0.84 (0.46, 1.53) | | | | |
| P trend |  | | | 1.00 | | |  | 0.50 |  | | | |  | 0.17 | | | | |
| Continuous variable |  | | |  | | |  |  |  | | | |  |  | | | | |
| Every 3 Claims |  | | | 0.95 (0.92, 0.99) | | |  | 0.99 (0.95, 1.03) |  | | | |  | 1.00 (0.96, 1.05) | | | | |

a. adjusting for sex, CCI, registry area, urbanization, income census index, education census index, diagnosis age and diagnosis year, tobacco use.

b. adjusting for sex, CCI, diagnosis age, diagnosis year.

c. adjusting for sex, registry area, urbanization, diagnosis year and tobacco use.

d. adjusting for sex, CCI, registry area, urbanization, income census index, education census index, diagnosis age and diagnosis year, histology, stage.

e. Proportional assumption model does not meet, use flexible parameter survival model.

f. NHW: Non-Hispanic White

g. HR: Hazard Ratio; CIs: Confidence Intervals

h. NSCLC: non-small cell lung cancer; SCLC: small cell lung cancer

i. Counts < 11 are not shown, Centers for Medicare & Medicaid Services (CMS) Cell Suppression Policy

**Supplementary Table 8. Fine-Gray Competing Risk Model for the Risk of CVD among Lung Cancer Patients, ANHPI and ANHPI Subgroup V.S. NHW**

|  | Heart Failure | Ischemic Heart Disease | | Stroke/Transient Ischemic Attack | |  |
| --- | --- | --- | --- | --- | --- | --- |
|  | HR (95% CIs) | | HR (95% CIs) | | HR (95% CIs) |  |
| NHW | 1 (ref) | | | | |  |
| Overall ANHPI | 0.70 (0.62, 0.78) | | 0.80 (0.69, 0.92) | | 1.00 (0.86, 1.18) |  |
| Chinese | 0.62 (0.50, 0.76) | | 0.67 (0.53, 0.85) | | 1.06 (0.81, 1.38) |  |
| Japanese | 0.56 (0.43, 0.73) | | 0.83 (0.62, 1.10) | | 1.19 (0.86, 1.65) |  |
| Filipino | 0.79 (0.63, 0.99) | | 0.81 (0.62, 1.07) | | 1.02 (0.74, 1.39) |  |
| Hawaiian | 0.77 (0.49, 1.22) | | 1.15 (0.66, 2.00) | | 0.83 (0.44, 1.56) |  |
| Korean | 0.75 (0.55, 1.01) | | 0.73 (0.49, 1.08) | | 0.50 (0.29, 0.85) |  |
| Vietnamese | 0.55 (0.41, 0.74) | | 0.82 (0.59, 1.15) | | 0.80 (0.53, 1.23) |  |
| Indian or Pakistani | 1.00 (0.66, 1.50) | | 1.41 (0.82, 2.40) | | 1.03 (0.61, 1.76) |  |
| Other Southeast Asian ^a^ | 1.03 (0.53, 1.99) | | 1.07 (0.48, 2.34) | | 1.06 (0.48, 2.32) |  |
| Pacific Islander | 1.62 (0.87, 3.02) | | 0.41 (0.16, 1.05) | | 1.09 (0.45, 2.63) |  |
| Other Asian ^b^ | 0.75 (0.53, 1.06) | | 0.77 (0.51, 1.17) | | 1.59 (1.09, 2.33) |  |
| a. Other Southeast Asian: including Laotian, Hmong, Kampuchean and Thai.  b. Other Asian: including Asian, NOS and Oriental, NOS.  c. ANHPI: Asian, Native Hawaiian, and Pacific Islander; NHW: Non-Hispanic White  d. HR: Hazard Ratio; CIs: Confidence Intervals.  e. Fine-Gray competing risk model, adjusting for matched pair and registry. | | | | | | |

**Supplementary Table 9. Fine-Gray Competing Risk Model for the Risk of CVD among Lung Cancer Patients, ANHPI Subgroup V.S. Chinese**

|  | Heart Failure | Ischemic Heart Disease | | Stroke/Transient Ischemic Attack |  |
| --- | --- | --- | --- | --- | --- |
|  | HR (95% CIs) | | HR (95% CIs) | HR (95% CIs) |  |
| Chinese | 1 (ref) | | | |  |
| Japanese | 1.08 (0.79, 1.48) | | 1.14 (0.81, 1.60) | 0.98 (0.67, 1.46) |  |
| Filipino | 1.34 (1.00, 1.78) | | 1.07 (0.77, 1.50) | 0.84 (0.57, 1.24) |  |
| Hawaiian | 1.27 (0.79, 2.03) | | 1.27 (0.74, 2.17) | 0.75 (0.38, 1.50) |  |
| Korean | 1.08 (0.76, 1.55) | | 0.97 (0.64, 1.46) | 0.52 (0.29, 0.92) |  |
| Vietnamese | 1.13 (0.79, 1.60) | | 1.25 (0.87, 1.80) | 0.81 (0.51, 1.29) |  |
| Indian or Pakistani | 1.99 (1.26, 3.15) | | 1.87 (1.12, 3.14) | 1.07 (0.58, 1.98) |  |
| Other Southeast Asian ^a^ | 1.72 (0.86, 3.46) | | 1.74 (0.77, 3.91) | 1.74 (0.74, 4.07) |  |
| Pacific Islander | 2.15 (1.15, 4.00) | | 0.69 (0.25, 1.88) | 0.94 (0.38, 2.36) |  |
| Other Asian ^b^ | 1.14 (0.78, 1.68) | | 1.07 (0.69, 1.66) | 1.35 (0.88, 2.09) |  |
| a. Other Southeast Asian: including Laotian, Hmong, Kampuchean and Thai.  b. Other Asian: including Asian, NOS and Oriental, NOS.  c. ANHPI: Asian, Native Hawaiian, and Pacific Islander; NHW: Non-Hispanic White  d. HR: Hazard Ratio; CIs: Confidence Intervals.  e. Fine-Gray competing risk model, adjusting for diagnosis year, diagnosis age, sex and registry. | | | | | |

| **Supplementary Table 10. Demographic and Clinical Characteristics of Chinese, Filipino, Indian and Pakistani, other Southeast Asian and Pacific Islander Lung Cancer Survivors** | | | | | |  |
| --- | --- | --- | --- | --- | --- | --- |
|  | Chinese lung cancer survivors (n=1042) | Filipino lung cancer survivors (n=652) | Indian and Pakistani lung cancer survivors (n=143) | other Southeast Asian lung cancer survivors ^f^ (n=54) | Pacific Islander lung cancer survivors (n=67) | |
|  | n (%) | n (%) | n (%) | n (%) | n (%) | |
| Sex | | | | |  |  |
| male | 531 (50.96) | 332 (50.92) | 83 (58.04) | 29 (53.70) | 33 (49.25) | |
| female | 511 (49.04) | 320 (49.08) | 60 (41.96) | 25 (46.30) | 34 (50.75) | |
| Age at diagnosis, year | | | | |  |  |
| 66-70 | 203 (19.48) | 164 (25.15) | >39 (>27.27) | 18 (33.33) | 25 (37.31) | |
| 71-75 | 256 (24.57) | 197 (30.21) | 41 (28.67) | 19 (35.19) | 14 (20.90) | |
| 76-80 | 263 (25.24) | 162 (24.85) | 37 (25.87) | * (*) | 18 (26.87) | |
| 81-85 | 201 (19.29) | 88 (13.50) | 15 (10.49) | * (*) | *(*) | |
| 86+ | 119 (11.42) | 41 (6.29) | <11 (<7.69) ^c^ | * (*) | * (*) | |
| Diagnosis year | | | | |  |  |
| 2000-2005 | 289 (27.74) | 174 (26.69) | 25 (17.48) | 12 (22.22) | 17 (25.37) | |
| 2006-2010 | 299 (28.69) | 173 (26.53) | 26 (18.18) | 14 (25.93) | 19 (28.36) | |
| 2011-2014 | 230 (22.07) | 168 (25.77) | 49 (34.27) | >17 (>31.48) | 18 (26.87) | |
| 2015-2017 | 224 (21.50) | 137 (21.01) | 43 (30.07) | <11 (<20.37) ^c^ | 13 (19.40) | |
| Charlson Comorbidity Index (CCI) at baseline | | | | |  |  |
| 0 | 421 (40.40) | 259 (39.72) | 56 (39.16) | 20 (37.04) | 25 (37.31) | |
| 1 | 386 (37.04) | 199 (30.52) | 44 (30.77) | 23 (42.59) | 21 (31.34) | |
| 2+ | 235 (22.55) | 194 (29.75) | 43 (30.07) | 11 (20.37) | 21 (31.34) | |
| Registry area | | | | |  |  |
| West | >945 (>90.69) | 598 (91.72) | 63 (44.06) | 44 (81.48) | 57 (85.07) | |
| Northeast | 73 (7.01) | 43 (6.60) | 52 (36.36) | * (*) | * (*) | |
| Midwest | <11 (<1.06) ^c^ | * (*) | 11 (7.69) | * (*) | * (*) | |
| South | 13 (1.25) | * (*) | 17 (11.89) | * (*) | * (*) | |
| follow up, year | | | | |  |  |
| ≥1-5 | 818 (78.50) | 524 (80.37) | >105 (73.43) | 45 (83.33) | >44 (65.67) | |
| ≥5-10 | 162 (15.55) | 97 (14.88) | 27 (18.88) | * (*) | 12 (17.91) | |
| ≥10 | 62 (5.95) | 31 (4.75) | <11 (<7.69) ^c^ | * (*) | <11 (16.42) ^c^ | |
| Urbanization ^a^ | | | | |  |  |
| Urban | >1031 (>98.94) | 620 (95.09) | >132 (>92.31) | >43 (>79.63) | >56 (>83.58) | |
| Rural | <11 (<1.06) ^c^ | 32 (4.91) | <11 (<7.69) ^c^ | <11 (<20.37) ^c^ | <11 (<16.42) ^c^ | |
| Education: proportion above college ^b^ (Census tract) | | | | |  |  |
| 0%-40% | 168 (16.12) | 107 (16.41) | * (*) | * (*) | 16 (23.88) | |
| < 40%-60% | 261 (25.05) | >246 (>37.73) | 42 (29.37) | 26 (48.15) | 25 (37.31) | |
| < 60%-80% | >393 (>37.72) | 232 (35.58) | 67 (46.85) | 13 (24.07) | 20 (29.85) | |
| < 80%-100% | 209 (20.06) | 56 (8.59) | 27 (18.88) | * (*) | *(*) | |
| Missing | <11 (<1.06) ^c^ | <11 (<1.69) ^c^ | * (*) | * (*) | *(*) | |
| Income (median income in census tract) | | | | |  |  |
| ≤40,000 | 245 (23.51) | 116 (17.79) | 13 (9.09) | 13 (24.07) | 15 (22.39) | |
| 40,000-60,000 | 252 (24.18) | >207 (>31.75) | 41 (28.67) | 21 (38.89) | 25 (37.31) | |
| 60,000-80,000 | 260 (24.95) | 173 (26.53) | 31 (21.68) | 12 (22.22) | 19 (28.36) | |
| >80,000 | >274 (>26.30) | 145 (22.24) | >47 (>32.87) | * (*) | * (*) | |
| Missing | <11 (<1.06) ^c^ | <11 (1.69) ^c^ | <11 (<7.69) ^c^ | * (*) | * (*) | |
| Tobacco use | | | | |  |  |
| Yes | <11 (<1.06) ^c^ | <11 (1.69) ^c^ | <11 (<7.69) ^c^ | <11 (<20.37) ^c^ | <11 (16.42) ^c^ | |
| No | >1031 (>98.94) | >641 (98.31) | >132 (>92.31) | >43 (>79.63) | >56 (>83.58) | |
| Sequence number | | | | |  |  |
| One primary only | 932 (89.44) | 590 (90.49) | 125 (87.41) | >43 (>79.63) | 54 (80.60) | |
| First of many primaries | 110 (10.56) | 62 (9.51) | 18 (12.59) | <11 (<20.37) ^c^ | 13 (19.40) | |
| Histology | | |  |  |  | |
| SCLC-Small cell | 38 (3.65) | 33 (5.06) | * (*) | * (*) | * (*) | |
| NSCLC-Adenocarcinoma | 688 (66.03) | >428 (>65.64) | 94 (65.73) | 32 (59.26) | 37 (55.22) | |
| NSCLC-Squamous cell | 124 (11.90) | 86 (13.19) | 22 (15.38) | * (*) | 15 (22.39) | |
| NSCLC-Large cell carcinoma | 23 (2.21) | <11 (<1.69) ^c^ | * (*) | * (*) | * (*) | |
| Other NSCLC | 109 (10.46) | 58 (8.90) | 12 (8.39) | * (*) | * (*) | |
| Unspecific lung cancer | 60 (5.76) | 36 (5.52) | * (*) | * (*) | * (*) | |
| Grade | | | | |  |  |
| Grade I | 93 (8.93) | 60 (9.20) | 17 (11.89) | 11 (20.37) | * (*) | |
| Grade II | 243 (23.32) | 134 (20.55) | 38 (26.57) | *(*) | 22 (32.84) | |
| Grade III | 218 (20.92) | 141 (21.63) | 23 (16.08) | 11 (20.37) | 17 (25.37) | |
| Grade IV | 26 (2.50) | 13 (1.99) | <11 (<7.69) ^c^ | * (*) | * (*) | |
| Not determined/stated/applicable | 462 (44.34) | 304 (46.63) | >54 (>37.76) | 23 (42.59) | 24 (35.82) | |
| Laterality | | | | |  |  |
| Right: origin of primary | 578 (55.47) | 391 (59.97) | >64 (>44.76) | >19 (35.19) | >27 (40.30) | |
| Left: origin of primary | 435 (41.75) | 245 (37.58) | 68 (47.55) | 24 (44.44) | 29 (43.28) | |
| Others ^d^ | 29 (2.78) | 16 (2.45) | <11 (<7.69) ^c^ | <11 (20.37) ^c^ | <11 (<16.42) ^c^ | |
| Stage | | | | |  |  |
| Localized | 293 (28.12) | 190 (29.14) | 49 (34.27) | 16 (29.63) | 22 (32.84) | |
| Regional | 279 (26.78) | 187 (28.68) | 43 (30.07) | 18 (33.33) | 21 (31.34) | |
| Distant | 470 (45.11) | 275 (42.18) | 51 (35.66) | 20 (37.04) | 24 (35.82) | |
| Radiation therapy (Radiation) | | |  |  |  | |
| Yes | 335 (32.15) | 253 (38.80) | 54 (37.76) | *(*) | 23 (34.33) | |
| No | >696 (66.79) | >388 (>59.51) | >78 (>54.55) | 42 (77.78) | >33 (>49.25) | |
| Unknown | <11 (<1.06) ^c^ | <11 (1.69) ^c^ | <11 (<7.69) ^c^ | * (*) | <11 (<16.42) ^c^ | |
| Chemotherapy | | | | |  |  |
| Yes | 477 (45.78) | 300 (46.01) | 56 (39.16) | 18 (33.33) | 32 (47.76) | |
| No/Unknown ^e^ | 565 (54.22) | 352 (53.99) | 87 (60.84) | 36 (66.67) | 35 (52.24) | |
| Surgery | | | | |  |  |
| Yes | 352 (33.78) | 202 (30.98) | 60 (41.96) | 17 (31.48) | 27 (40.30) | |
| No | 690 (66.22) | 450 (69.02) | 83 (58.04) | 37 (68.52) | 40 (59.70) | |

a. Urban is defined as counties of metropolitan areas (code 0-3 of Rural-Urban Continuum/Beale code); Rural is defined as cities of non-metropolitan areas (code 4-9 of Rural-Urban Continuum/Beale code).

b. Including some college and at least 4 years of college.

c. Counts < 11 are not shown, Centers for Medicare & Medicaid Services (CMS) Cell Suppression Policy

d. Others include: Not a paired site; Only one side involved, right or left origin unspecified; Bilateral involvement, lateral origin unknown; Stated to be single primary; Paired site

e. The original dataset combined No and Unknown together for Chemotherapy.

f. Other Southeast Asian: including Laotian, Hmong, Kampuchean and Thai.

g. ANHPI: Asian, Native Hawaiian, and Pacific Islander; NHW: Non-Hispanic White.

*. Hidden because counts < 11, Centers for Medicare & Medicaid Services (CMS) Cell Suppression Policy

Supplementary Figures

Supplementary Figure 1. Kaplan-Meier Curve for Lung Cancer Patients at Risk of Heart Failure


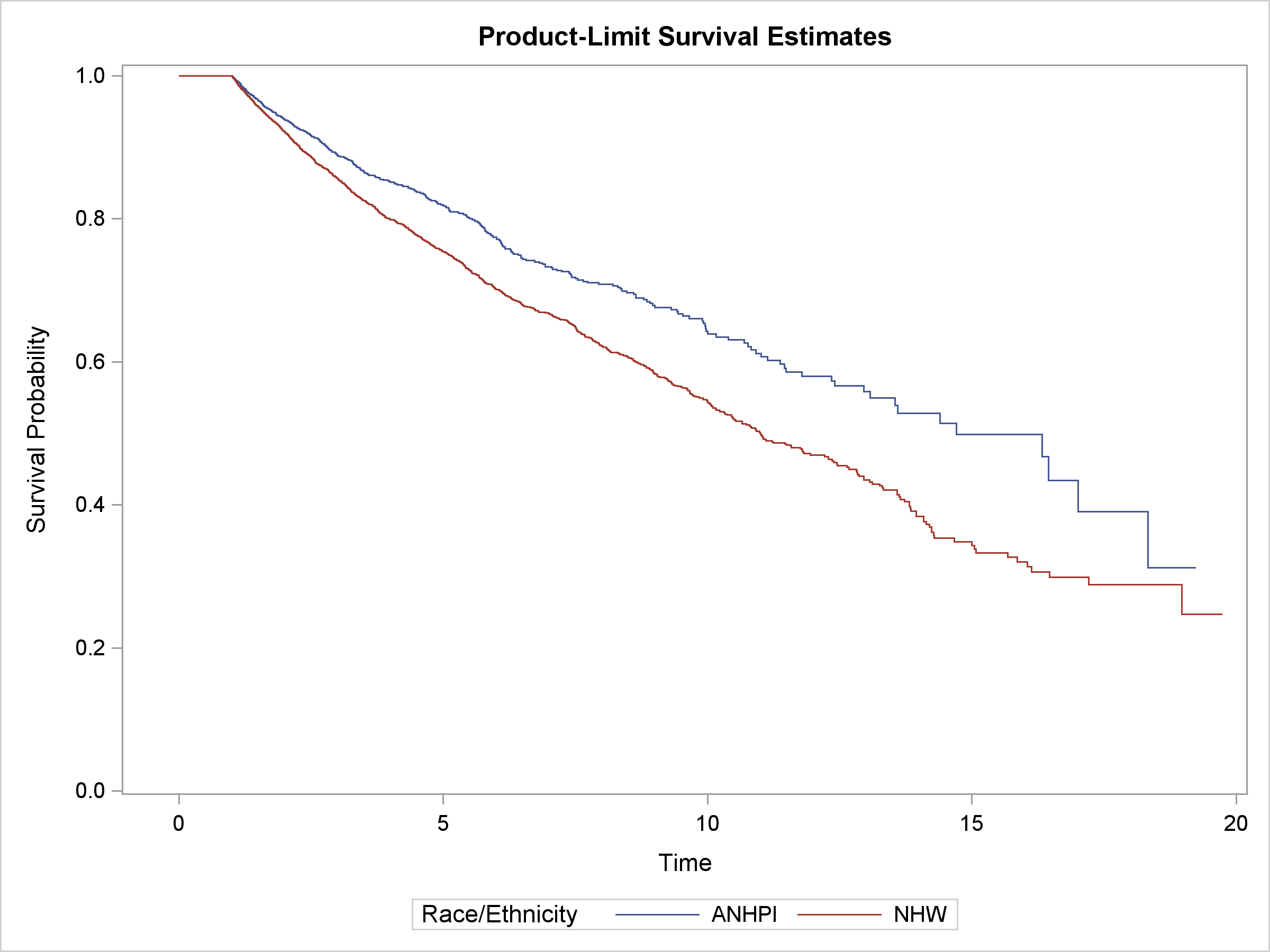


Supplementary Figure 2. Kaplan-Meier Curve for Lung Cancer Patients at Risk of Ischemic Heart Disease


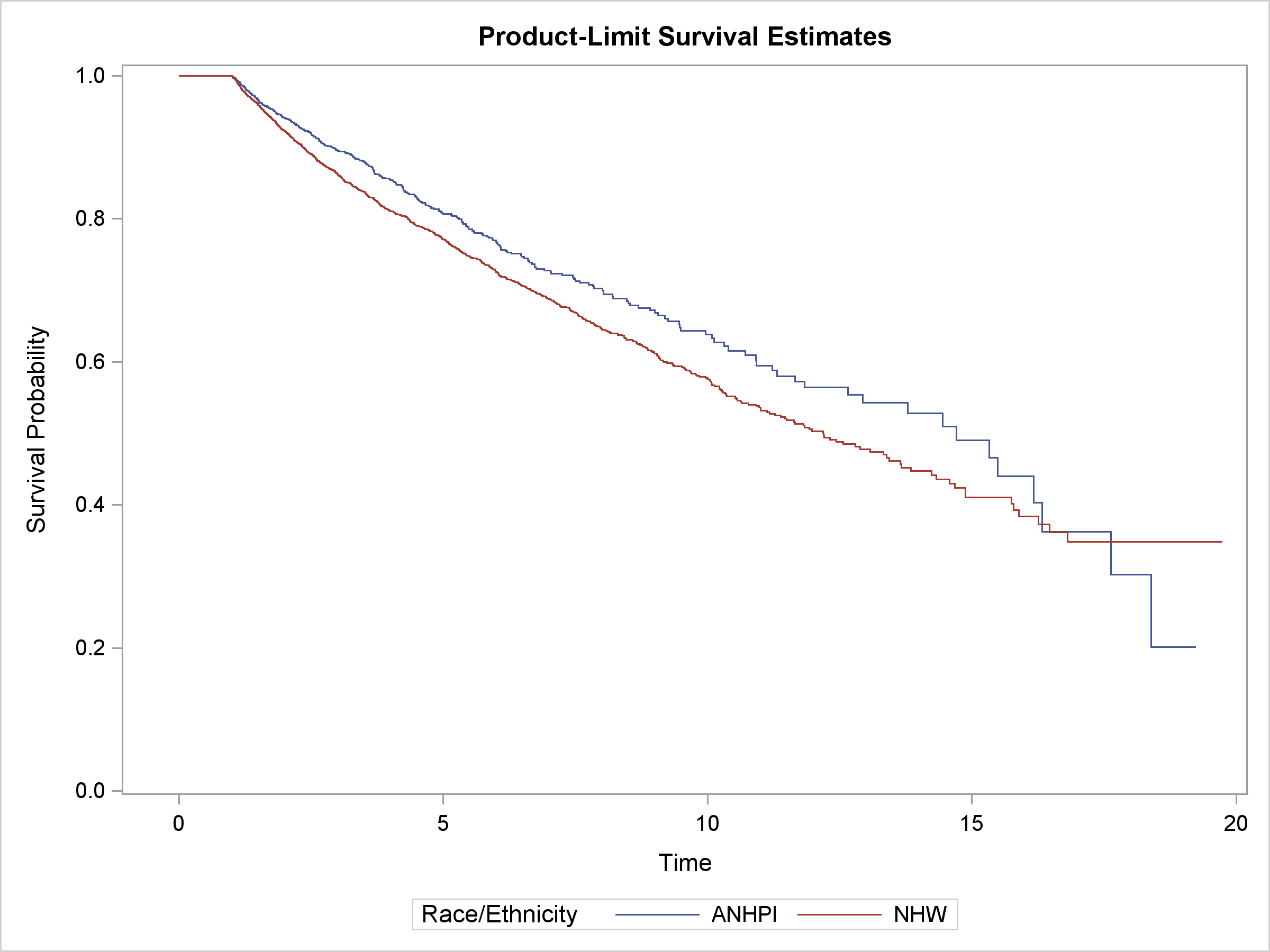


Supplementary Figure 3. Kaplan-Meier Curve for Lung Cancer Patients at Risk of Stroke/ Transient Ischemic Attack


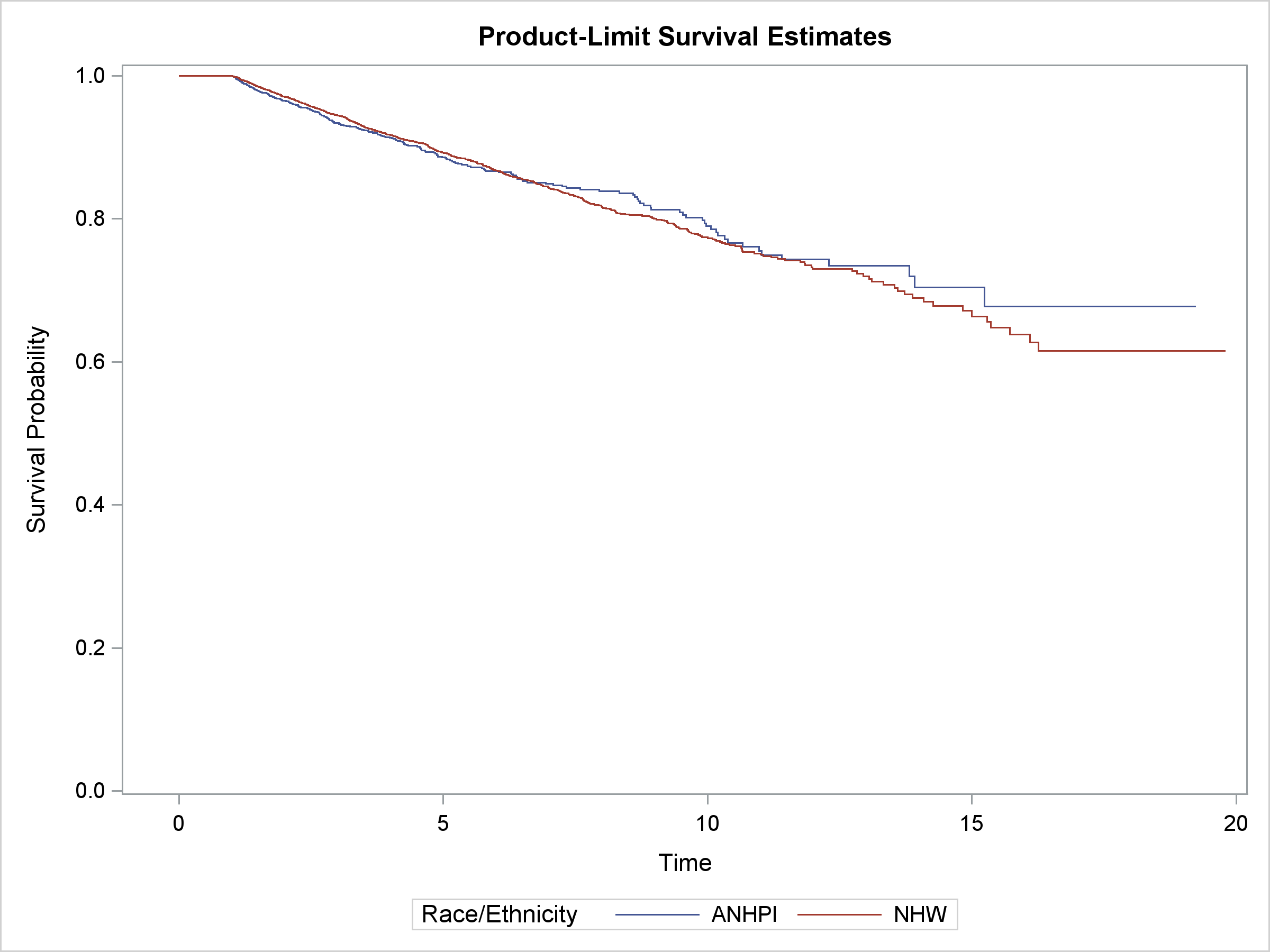


Supplementary Figure 4. Directed Acyclic Graph (DAG) from Race/Ethnicity to CVD outcomes.


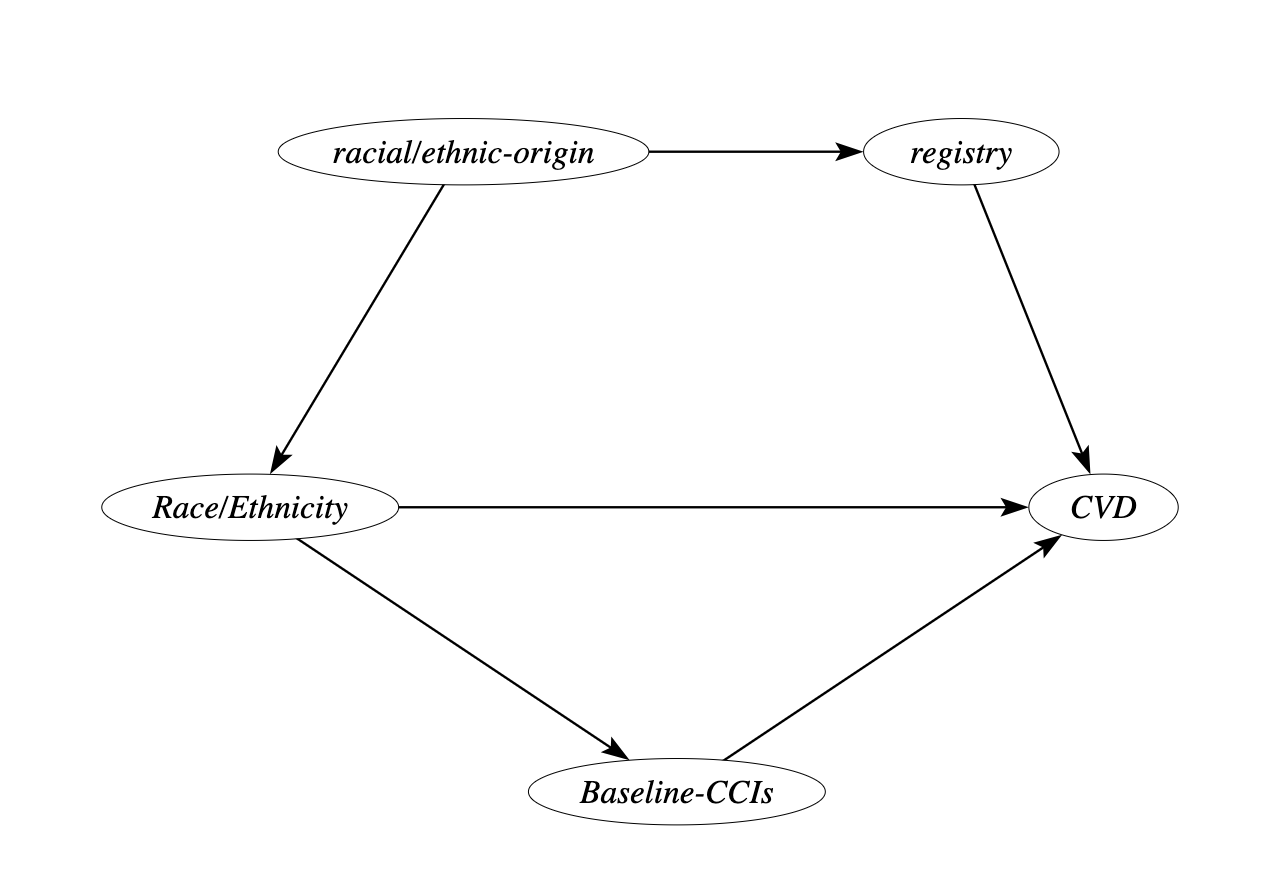


Supplementary Figure 5. Cumulative Incidence Curve for Lung Cancer Patients at Risk of Heart Failure, Considering Competing Risk of Death


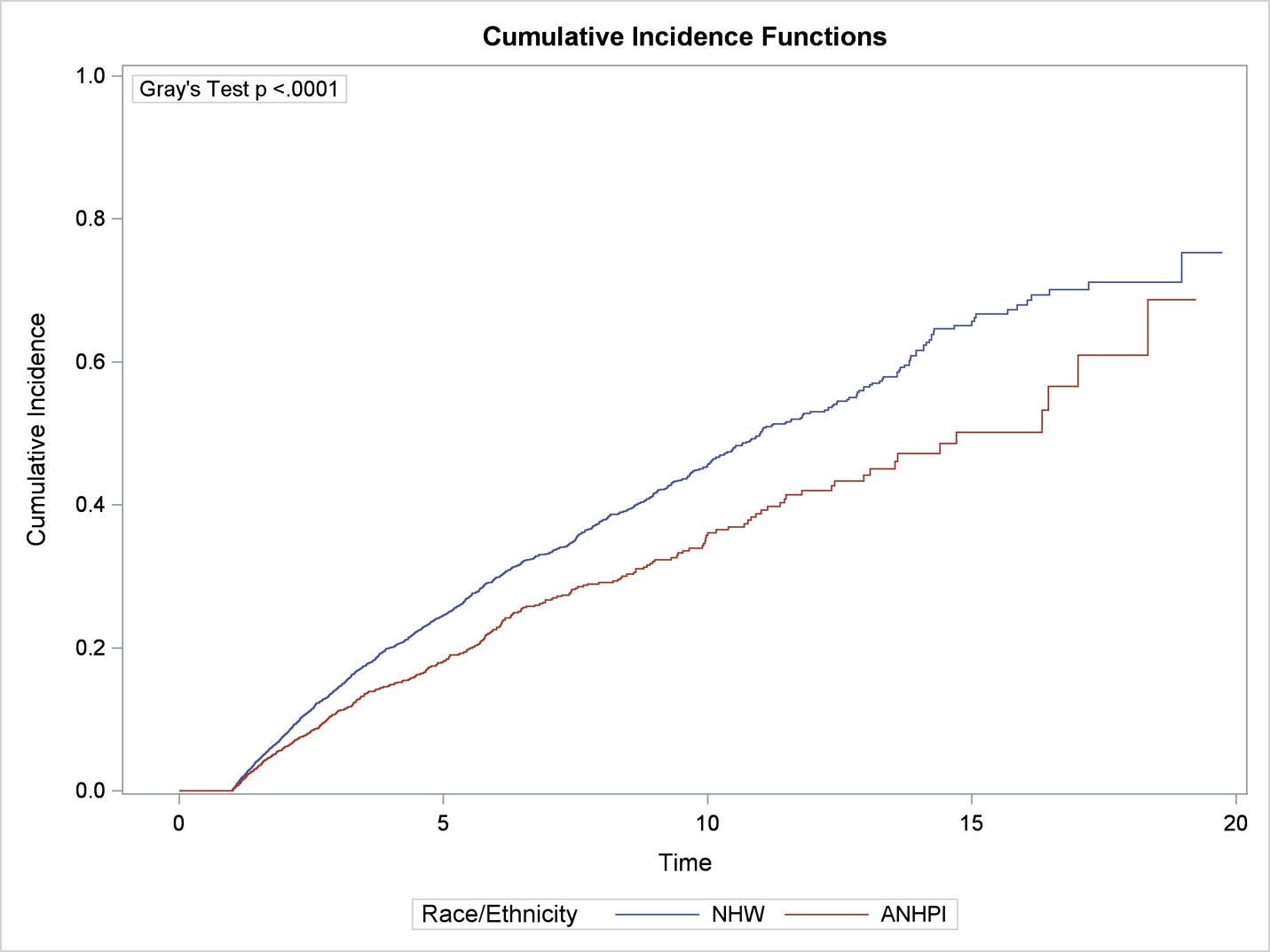


Supplementary Figure 6. Cumulative Incidence Curve for Lung Cancer Patients at Risk of Ischemic Heart Disease, Considering Competing Risk of Death


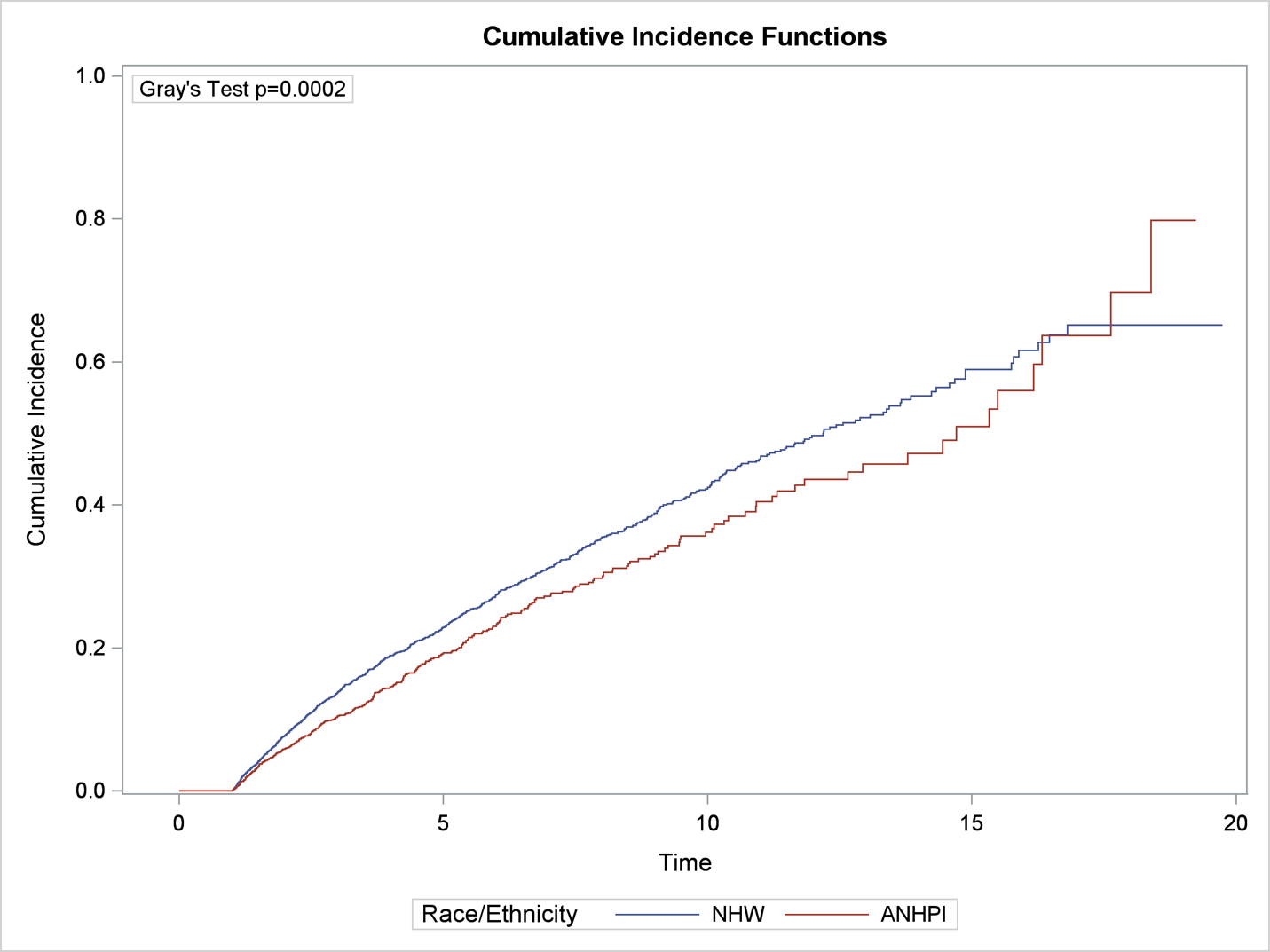


Supplementary Figure 7. Cumulative Incidence Curve for Lung Cancer Patients at Risk of Stroke/ Transient Ischemic Attack, Considering Competing Risk of Death


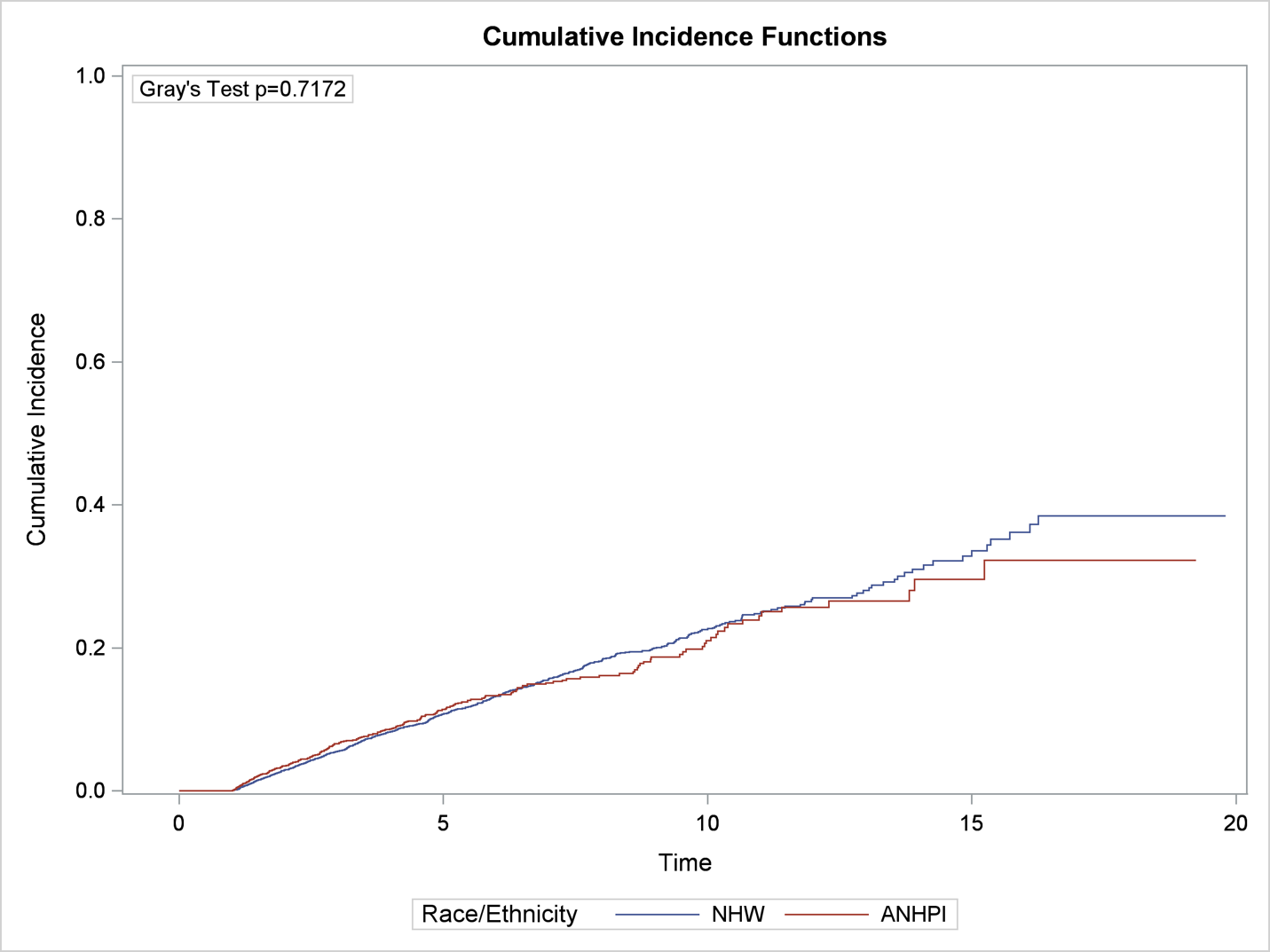

Supplement: Supplementary file 1 — Appendix S1 [file CAM4-14-e70702-s001.docx]
